# Genomic surveillance of Salmonella enterica serotype Minnesota strains from poultry products imported into South Africa

**DOI:** 10.1099/mgen.0.001633

**Published:** 2026-02-05

**Authors:** Vishnu Raghuram, Thendo Mafuna, Vignesh Ramnath, Hadrien Gourlé, Josefin Blom, Kudakwashe Magwedere, Laura M. Carroll, Itumeleng Matle

**Affiliations:** 1 Department of Clinical Microbiology, SciLifeLab, Umeå University, Umeå, Sweden; 2 Laboratory for Molecular Infection Medicine Sweden (MIMS), Umeå University, Umeå, Sweden; 3 Umeå Centre for Microbial Research (UCMR), Umeå University, Umeå, Sweden; 4 Integrated Science Lab (IceLab), Umeå University, Umeå, Sweden; 5 Department of Biochemistry, University of Johannesburg, Auckland Park, South Africa; 6 Unaffiliated Global Health Security Research Scholar, Pretoria, South Africa; 7 Department of Agriculture and Animal Health, University of South Africa, Science Campus, Florida, South Africa

**Keywords:** antimicrobial resistance, beta-lactamase, CTX-M-8, poultry, *Salmonella *Minnesota, whole-genome sequencing

## Abstract

*Salmonella enterica* subspecies *enterica* serotype Minnesota (*S*. Minnesota) has recently emerged as a predominant serotype in poultry farming operations. Genomic surveillance efforts concentrated primarily in Europe have been used to evaluate food safety risks associated with *S*. Minnesota in imported poultry/poultry products. However, the burden imposed by *S*. Minnesota on consumers in sub-Saharan Africa is not understood. Here, we used whole-genome sequencing to characterize 36 *S*. Minnesota strains from raw poultry imported into South Africa, specifically (i) 11 strains isolated at port of entry and (ii) 25 strains from imported poultry in South African supermarkets. While all 36 strains belonged to the same sequence type (ST548), multiple ST548 lineages were present among poultry products. Comparison of the 36 strains sequenced here to all publicly available, high-quality ST548 genomes (*n*=460, from Enterobase) identified several public genomes differing by <30 core SNPs, including strains isolated previously from South American poultry imported into the UK. Notably, a cluster consisting of 14 highly similar genomes sequenced here (0 core SNPs) uniquely possessed *bla*_CTX-M-8_. A search of plasmids in public databases, alongside antimicrobial resistance (AMR) genes from >1.9 million bacterial genomes, revealed that this cluster harboured *bla*_CTX-M-8_ on an IncI1 plasmid-like region, which we hypothesize was acquired recently, from *Escherichia coli*. Overall, our study provides insight into the intercontinental dissemination of *S*. Minnesota and its associated AMR determinants via the global poultry trade.

**Impact Statement**

Raw poultry exports have allowed the dissemination of *Salmonella enterica* subspecies *enterica* serotype Minnesota (*S*. Minnesota) internationally, and several countries (primarily in Europe) have used whole-genome sequencing (WGS) to characterize * S*. Minnesota strains from imported poultry. While many African countries also import significant quantities of poultry, very little is known about *S*. Minnesota in South Africa, let alone sub-Saharan Africa. Here, we detect multiple *S*. Minnesota ST548 lineages at two control points in South Africa (port-of-entry and supermarkets), including emerging AMR lineages. Notably, we found that all *S*. Minnesota genomes from South Africa were closely related to poultry/food-associated *S*. Minnesota genomes from the UK or South America. Recent WGS-based studies from the UK have posited that *S*. Minnesota from poultry is unlikely to cause illness, should it reach UK consumers. While no links to clinical cases in South Africa were observed here, this could be due to data gaps, as the vast majority of *Salmonella* WGS efforts are concentrated in Europe and North America. Our study highlights the important role that genomic surveillance plays in mitigating food safety risks associated with the global agro-food trade and showcases the importance of local pathogen surveillance initiatives.

## Data Summary

Whole-genome sequencing data generated in this study are available under NCBI (National Center for Biotechnology Information) BioProject accession PRJNA1230142. NCBI BioSample and Sequence Read Archive accessions for newly sequenced genomes, as well as for the publicly available genomes used in this study, are available in the Supplementary Material. All code for running software is described in the article, and additional data analysis code is available via GitHub (https://github.com/VishnuRaghuram94/SEPI). Tables S1–S3 are available alongside the article. All larger supplementary datasets (Datasets S1–S19) and intermediate files are available via doi: 10.5281/zenodo.15063661.

## Introduction

Foodborne zoonotic pathogen *Salmonella enterica* imposes a massive burden on global public health [1,2]. Each year, *Salmonella* is estimated to cause 93.8 million cases of gastroenteritis globally, the vast majority of which (>85%) are attributed to the consumption of contaminated food [3]. More specifically, contaminated poultry is widely regarded to be a major source of *Salmonella* illnesses [1,4,6]. In the USA, chicken and turkey products have been estimated to account for nearly a quarter of foodborne *Salmonella* illness cases [7,8].

While poultry can serve as a reservoir for a range of *Salmonella* serotypes [9,11], *Salmonella enterica* subspecies *enterica* serotype Minnesota (*S*. Minnesota), though a relatively rare serotype globally, has recently emerged as a serotype of concern in Brazilian poultry farming operations [4,12, 13]. It has been hypothesized that a combination of factors contributed to *S*. Minnesota’s rapid rise in Brazil, including the 2003 introduction of a vaccine against *Salmonella* Enteritidis (the predominant serotype in Brazilian poultry at the time), antimicrobial use and antimicrobial resistance (AMR) [4,12, 14, 15].

From a genomic surveillance perspective, a great deal of effort has gone into monitoring the spread of *S*. Minnesota and its associated AMR determinants in Brazil [4,13, 16, 17]. However, due to Brazil’s status as a leading global poultry exporter, * S*. Minnesota can be disseminated to other countries via imported Brazilian poultry/poultry products [4,12]. As such, several other countries have employed whole-genome sequencing (WGS) to characterize *S*. Minnesota strains from Brazilian poultry imports [4,12, 18, 19]. These efforts have been largely confined to Europe (e.g. the UK and Portugal) [4,18] and, only recently, Saudi Arabia [12]. Very little is known about *S*. Minnesota in sub-Saharan Africa, and WGS-based studies of *S*. Minnesota from imported poultry have not been conducted in Africa, despite the fact that 16% of all chicken and 35% of all turkey exports from Brazil are to Africa (~840,000 metric tonnes in 2023) [20].

Port-of-entry pathogen surveillance efforts in South Africa have highlighted the potential food safety risks associated with imported poultry products [21]. Given that (i) it is not uncommon to isolate *S*. Minnesota from imported poultry products tested at South African port-of-entry [21] and (ii) Brazil has been the largest exporter of poultry meat to South Africa since 2001 [22], poultry products imported from Brazil may serve as a vehicle by which *S*. Minnesota can enter South Africa’s domestic food system. However, the extent to which this has occurred has not been studied. Here, we used WGS to characterize 36 *S*. Minnesota strains isolated from raw poultry imported into South Africa from Brazil [referred to as ‘SEPI’ genomes (*Salmonella enterica* Poultry Imports)]. By comparing our novel genomes to hundreds of relevant high-quality, publicly available *S*. Minnesota genomes from around the world, we provide insight into the dissemination of *S*. Minnesota and its associated AMR determinants in South Africa.

## Methods

### Study design and source of isolates

This retrospective study was conducted using laboratory-confirmed *Salmonella* isolates stored at the Agricultural Research Council–Onderstepoort Veterinary Research (ARC-OVR) General Bacteriology Laboratory. The isolates were recovered from culture samples obtained during routine-controlled inspections at ports of entry (POEs), routine diagnostic services and research studies at supermarkets. Sampling was not part of this study; however, isolates were sent to the ARC-OVR General Bacteriology Laboratory following the protocol recommended in Veterinary Procedural Notification 56, accompanied by all necessary documentation and metadata. This included details such as the source of isolation, country or location of origin and date of isolation or sample collection, among other relevant information.

### Sample isolation, DNA extraction and WGS

Bacterial isolation and identification were carried out following the guidelines outlined in ‘Microbiology of the Food Chain: Horizontal Method for the Detection, Enumeration and Serotyping of *Salmonella*’ (ISO 6579-1:2017) [23]. Briefly, genomic DNA was extracted from overnight cultures using the High Pure PCR Template Preparation Kit (Roche, Germany) in accordance with the manufacturer’s instructions. WGS of the isolates was conducted at the Biotechnology Platform of the Agricultural Research Council, Onderstepoort, South Africa. DNA libraries were prepared using TruSeq DNA Library Preparation Kits (Illumina, San Diego, CA, USA) and sequenced on the HiSeq 2500 platform (Illumina, San Diego, CA, USA), according to the manufacturer’s instructions. Thirty-six independent isolates were used in this study.

### Quality control, pre-processing and assembly of WGS data

Trimming/filtering of raw Illumina paired-end reads was performed using fastp v0.23.4 with default parameters [24]. The resulting trimmed reads were assembled using Shovill v1.1.0 with the ‘--assembler skesa’ parameter (otherwise default parameters; https://github.com/tseemann/shovill) [25]. Quality control of the resulting assemblies was performed using (i) QUAST v5.3.0 [26], with the complete *S*. Minnesota sequence type 548 (ST548) str. ATCC 49284 genome as the reference (‘-r’ option, NCBI [National Center for Biotechnology Information] Assembly accession GCA_000486855.2; see the ‘*In silico* serotyping and sequence typing’ section for sequence typing details) [27] (ii) CheckM v1.2.2 [28], using the ‘lineage_wf’ workflow with default parameters (Dataset S1, available in the online Supplementary Material).

### *In silico* serotyping and sequence typing

*In silico* serotyping of assembled genomes (see the ‘Quality control, pre-processing, and assembly of WGS data’ section) was performed using (i) SISTR v1.1.1 (default parameters) [29] and (ii) SeqSero2 v1.3.1 (‘-m k -t 1’; otherwise default parameters) [30]. *In silico* seven-gene multi-locus sequence typing (MLST) was performed using mlst v2.9 (https://github.com/tseemann/mlst) with default parameters (Dataset S1), assigning all genomes sequenced here to ST548.

### Acquisition of publicly available WGS data and metadata

All publicly available ST548 genomes (see the ‘*In silico* serotyping and sequence typing’ section) and associated metadata were retrieved from Enterobase (*n*=518 ST548 genomes, accessed on 29 July 2025) [31,32]. Samples were included only if (i) they were associated with a BioProject [33], (ii) a year of isolation was recorded and (iii) SISTR1 serotype=SeqSero2 serotype=‘Minnesota’. Out of these 518 samples, 347 had assemblies available in NCBI GenBank (accessed on 29 July 2025), from which only assemblies with (i) N50 >50 Kbp, (ii) number of contigs <500 and (iii) species percentage≥85% were retained (assembly statistics obtained via Enterobase and downloaded from GenBank using NCBI datasets v15.29.0). The raw reads for the remaining 171 samples without an associated GenBank assembly were downloaded from the Sequence Read Archive (SRA) (accessed on 29 July 2025) using fasterq-dump v3.0.7 and assembled using the workflow described in the ‘Quality control, pre-processing, and assembly of WGS data’ section. Assembly statistics were calculated using QUAST v5.3.0 and filtered with criteria identical to the GenBank assemblies (N50 >50 Kbp, <500 contigs, ≥85% aligned to *S. enterica* ATCC 49284 reference). This resulted in a total of 461 publicly available genomes (294 GenBank+167 SRA).

To conduct a rough assessment of overall genome similarity among the 461 publicly available ST548 genomes, a Mash distance-based tree [34] was constructed using mashtree v1.4.6 [35], using ‘--genome-size 4769836’ (i.e. the average length of the 461 publicly available genomes). Inspection of the resulting Mash distance-based tree revealed a single outlier genome (NCBI Assembly accession GCA_011665355.1); this outlier genome was relatively distant from all other publicly available ST548 genomes and was thus not included in further analyses (Fig. S1). The remaining 460 publicly available ST548 genomes (Dataset S2) were combined with the 36 newly sequenced genomes (see the ‘Sample isolation, DNA extraction, and WGS’ section) to form the full dataset used in subsequent analyses (*n*=496 total ST548 genomes, referred to hereafter as the ‘full ST548 dataset’; Table S1).

### AMR determinant and plasmid replicon detection

All 496 genomes in the full ST548 dataset (see the ‘Acquisition of publicly available WGS data and metadata’ section) were screened for AMR determinants using AMRFinderPlus v3.12.8 [36,37], with the ‘-O Salmonella --plus’ options and database version 2024-05-02.2 (otherwise default parameters, 50% coverage and 90% amino acid identity cutoff; Dataset S3). ABRicate v1.0.1 (https://github.com/tseemann/abricate) was used with the PlasmidFinder database (‘--db plasmidfinder’, otherwise default parameters– 80% coverage and 80% nucleotide identity cutoff) to identify plasmid replicons in each genome (PlasmidFinder version 2023-Nov-4; Dataset S4) [38].

### Pan-genome analysis

All 496 genomes in the full ST548 dataset (see the ‘Acquisition of publicly available WGS data and metadata’ section) were annotated using Bakta v1.8.2 with database version 5.0 [39]. The resulting .gff3 files were used as input for the pan-genome estimation software Panaroo v1.3.4 [40] with the following parameters: ‘-f 0.5 --core_threshold 0.95 --remove-invalid-genes --clean-mode strict -a pan’. The resulting ‘gene_presence_absence.Rtab’ file (Dataset S5) was imported into R v4.4.0 (https://www.R-project.org/) and filtered to contain only intermediate genes (i.e. genes present in <95% and >5% of the population). The intermediate gene presence/absence matrix was supplied as input to the R package umap v0.2.10.0 (https://CRAN.R-project.org/package=umap), and the resulting Uniform Manifold Approximation and Projection for Dimension Reduction (UMAP) [41] was visualized using ggplot2 v3.5.1 [42].

### Core SNP calling and ML phylogeny construction for the full ST548 dataset

A core genome alignment was generated using Snippy v4.6.0 with the complete *S*. Minnesota sequence type 548 (ST548) str. ATCC 49284 genome as the reference (NCBI Assembly accession GCA_000486855.2) and otherwise default parameters (https://github.com/tseemann/snippy). Recombinant regions in the core genome alignment were masked using Gubbins v3.3.1 [43] with the options ‘--tree-builder iqtree-fast --first-tree-builder iqtree-fast’ (otherwise default parameters). snp-sites v2.5.1 [44] with ‘-c’ (otherwise default parameters) was used to extract variable sites in the recombination-masked alignment, resulting in a final recombination-free core SNP alignment. The final recombination-free core SNP alignment was used to calculate all-vs-all pairwise SNP distances using snp-dists v0.8.2 with default parameters (https://github.com/tseemann/snp-dists; Dataset S6). The above workflow was repeated to generate all-vs-all pairwise SNP distances for the 36 newly sequenced SEPI genomes alone, with publicly available genomes omitted (using SEPI genome 101 N as the reference; Dataset S7). Pairwise SNP distances were visualized using the R package pheatmap v1.0.12 (https://CRAN.R-project.org/package=pheatmap) with average linkage hierarchical clustering performed by hclust (from R package stats v4.3.2).

Phylogenetic inference was performed in IQ-TREE v2.2.5 [45], using the final recombination-free core SNP alignment for the full ST548 dataset as input and the following parameters: (i) the GTR+R nucleotide substitution model [46,48]; (ii) 1,000 ultrafast bootstrap replicates [49]; (iii) the number of constant sites in the alignment (obtained using snp-sites v2.5.1 with the ‘-C’ option), specified using the ‘-fconst’ parameter (i.e. ‘1092244,1194114,1193741,1094362’ for A, C, G and T, respectively); (iv) ‘--seed’ was set to 1,000. The resulting unrooted maximum likelihood (ML) phylogeny was imported into R v4.4.0, using the package ggtree v3.11.1 [50] and subsequently midpoint rooted using the ‘midpoint_root’ function in phytools v2.1-1 (referred to hereafter as the ‘midpoint-rooted ML phylogeny’; Fig. S3) [51].

Two rooted, time-scaled phylogenies were additionally constructed using Rlsd2 v2.4.4 (https://github.com/tothuhien/Rlsd2) [52], with the unrooted ML phylogeny from IQ-TREE supplied as input, and tip dates corresponding to the year of isolation reported for each genome: (i) a fixed-rate ML phylogeny, with the options ‘seqLen=4708764, estimateRoot = ‘as’, confidenceInterval=1,000, givenRate=1.27e-7’ (Fig. S4) [13], and (ii) a LSD2-estimated-rate phylogeny, constructed as described in (i), but without supplying ‘givenRate=1.27e-7’ to Rlsd2 (Fig. S5). Subsequent analysis of the unrooted ML phylogeny in TempEst v1.5.3 [53] using the ‘best-fitting root’ option. All three ML phylogenies (i.e. the midpoint-rooted, LSD2-fixed-rate and LSD2-estimated-rate ML phylogenies) are provided (10.5281/zenodo.15063661).

### Core SNP calling and ML phylogeny construction for the high-AMR group

Additional ML phylogenies were constructed for a well-supported clade within the full ST548 dataset, which harboured many AMR determinants (i.e. the ‘high-AMR group’). Briefly, genome names for members of the high-AMR group were extracted from the larger ST548 phylogeny using the ‘mrca’ function in the R package ape v5.7-1 (*n*=241 high-AMR group genomes) [54]. The Snippy/Gubbins/snp-sites pipeline described above was used to identify core SNPs among all 241 high-AMR group genomes (using ATCC 49284 as the reference; see the ‘Core SNP calling and phylogeny construction’ section). The resulting recombination-free core SNPs were then used to build (i) midpoint-rooted (Fig. S6A) and (ii) LSD2-estimated-rate ML phylogenies (Fig. S6B) as described above (see the ‘Core SNP calling and phylogeny construction’ section).

### Identification of plasmid-mediated AMR genes

To determine which AMR genes were likely harboured on plasmids, the ‘end-to-end’ command in geNomad v1.7.1 [55] with ‘--enable-score-calibration’ was used to classify all contigs in the full ST548 dataset as ‘chromosomal’, ‘plasmid’ or ‘viral’ (see the ‘AMR determinant and plasmid replicon detection’ section; Dataset S8). ‘Aggregated-classification’ scores were compared between AMR gene-harbouring (AMR+) contigs and contigs without AMR genes (AMR-; as determined by AMRFinderPlus) using the Kruskal–Wallis test in R (‘kruskal.test’ function). The ‘calibrated-aggregated-classification’ scores for the AMR+contigs were then grouped by AMR gene and plotted in R using ggplot2 v3.5.1.

The plasmid database PLSDB version ‘2024_05_31_v2’ [56] was downloaded and a non-redundant version of the database was constructed using MMSeqs2 ‘easy-cluster’ (version 6f45232ac8daca14e354ae320a4359056ec524c2). This non-redundant PLSDB database was then converted into a blast v2.15.0 database using makeblastdb [57]. SeqKit v2.7.0 [58] was used to extract AMR+contigs from the corresponding assemblies, and the contigs were then queried against the non-redundant PLSDB blast database using megablast (‘blastn’ command with default parameters). Contigs ≥1,000 bp that aligned to plasmid sequences in the PLSDB database at >95% coverage and >95% identity were considered to be candidate AMR gene-harbouring plasmids (Dataset S9). AMRFinderPlus, using the database version 2024-05-02.2 (otherwise default parameters), was also run on the non-redundant PLSDB sequences (Dataset S10).

### Analysis of *bla*_CTX-M-8_-harbouring contigs in ZA SNP cluster 0

Beta-lactamase *bla*_CTX-M-8_ was detected in several identical ST548 genomes sequenced here (referred to hereafter as ‘ZA SNP cluster 0’ genomes; described in detail in the ‘Results’ section). All *bla*_CTX-M-8_-harbouring (*bla*_CTX-M-8_+) genomes underwent further investigation. Specifically, all *bla*_CTX-M-8_+ contigs matched a ∼90 Kbp IncI pST113 plasmid from *Escherichia coli* in the non-redundant PLSDB blast database with >95% coverage and >95% identity (NCBI Nucleotide accession CP134355.1; see the ‘Identification of plasmid-mediated AMR genes’ section; Dataset S9). The accession number CP134355.1 was supplied as the query for an NCBI Nucleotide blast search against the ‘core_nt’ database, using ‘blastn’ as the selected programme and the search limited to organism ‘Salmonella enterica (taxid:28901)’ (https://blast.ncbi.nlm.nih.gov/Blast.cgi, search conducted on 6 October 2025; Dataset S11) [59]. Aligned sequences for all hits were downloaded and underwent AMR determinant detection using AMRFinderPlus with the ‘-O Salmonella --plus’ options and database version 2024-05-02.2 (otherwise default parameters). This was done to identify similar plasmids in *S. enterica* and to determine if the hits also contained *bla*_CTX-M-8_. The only *bla*_CTX-M-8_+ hits were in two plasmids, both having >99% identity and >99% query coverage to the IncI pST113 plasmid from *E. coli*: (i) a~90 Kbp plasmid previously identified in *S*. Mbandaka (NCBI Nucleotide accession CP146619.1) [60] and (ii) a~150 Kbp plasmid sequence from *S*. Enteritidis (NCBI Nucleotide accession CP183502.1; Dataset S12). As the *S*. Mbandaka complete plasmid sequence was of similar size to the *E. coli* IncI plasmid (both ~90 Kbp) and associated with a publication as of 6 [7,60], it was chosen as the reference sequence for further analysis.

### Reconstruction of *bla*_CTX-M-8_ + plasmid from ZA SNP cluster 0 genomes

Trimmed forward and reverse reads from each ZA SNP cluster 0 genome (see the ‘Analysis of *bla*_CTX-M-8_-harbouring contigs’ section) were fed to the ‘plasmidspades’ command in SPAdes v4.0.0 [61] with the ‘--only-assembler’ parameter. The resulting ‘contigs.fasta’ file for each plasmid assembly, the *S*. Mbandaka *bla*_CTX-M-8_+ IncI plasmid (NCBI Nucleotide accession CP146619.1) and the *E. coli bla*_CTX-M-8_+ IncI plasmid (NCBI Nucleotide accession CP134355.1) were annotated using Bakta v1.8.2 (see the ‘AMR determinant and plasmid replicon detection’ and ‘Analysis of *bla*_CTX-M-8_-harbouring contigs’ sections). We also ran Panaroo v1.3.4 on all assembled plasmid components as described in the ‘Pan-genome analysis’ section to compare gene composition across plasmids (Dataset S13). Plasmid comparison and visualization were performed using Clinker v0.0.31 [62] with the ‘-i=0.95’ option using the Bakta .gbk files for plasmids reconstructed from SEPI genome 290 and the reference plasmids CP146619.1 and CP134355.1. For visualization, contigs from the genome 290 plasmidSPAdes assembly that did not align to the *S*. Mbandaka/*E. coli* reference IncI plasmids were excluded from the figure. ABRicate v1.0.1 was used with the PlasmidFinder database to detect plasmid replicons in the plasmidSPAdes contigs as described above (see the ‘AMR determinant and plasmid replicon detection’ section; Dataset S14).

### Read mapping against *bla*_CTX-M-8_+ plasmids

To identify *bla*_CTX-M-8_+ plasmids, AMRFinderPlus with database version 2024-05-02.2 (otherwise default parameters) was run on the non-redundant PLSDB sequences (Dataset S10; see the ‘Identification of plasmid-mediated AMR genes’ section). Trimmed reads from each of the SEPI genomes, as well as the ST548 SRA set genomes (i.e. samples for which raw reads were downloaded; see the ‘Acquisition of publicly available WGS data and metadata’ section), were independently aligned against CP134355.1, CP146619.1 and every other *bla*_CTX-M-8_+ plasmid in the non-redundant PLSDB database (*n*=21). Coverage metrics were calculated with bbmap v39.06, samtools v1.20 and bedtools v2.31.0 using the following workflow - ‘bbmap.sh nodisk=t ref=$plasmid.fna in=$R1.fq in2=$R2.fq out=stdout | samtools view -bS | samtools sort | bedtools genomecov -ibam stdin -bga > $bedgraph.bg’. Normalized read counts for each genome were obtained by dividing the total number of reads mapped by the number of reads mapped to *rpoD* (measured using the same bbmap, samtools and bedtools workflow). The reference sequence for *rpoD* was retrieved from Panaroo (‘pan_genome_reference.fa’; see the ‘Pan-genome analysis’ section). We also performed read mapping and coverage metric calculation for the ten most abundant *bla*_CTX-M-8_- plasmids in our dataset as a control (Dataset S9).

Collectively, this analysis gave us (i) the coverage and normalized number of mapped reads for *bla*_CTX-M-8_+ and *bla*_CTX-M-8_- genomes when mapped to *bla*_CTX-M-8_+ plasmids and (ii) the coverage and normalized number of mapped reads for *bla*_CTX-M-8_+ and *bla*_CTX-M-8_- genomes when mapped to *bla*_CTX-M-8_- plasmids (Dataset S15). We then sought to test if coverage and the number of mapped reads to *bla*_CTX-M-8_+ plasmids were dependent on genomic *bla*_CTX-M-8_ status. For each combination of genomic and plasmid *bla*_CTX-M-8_ status, we visualized the distribution of coverage and normalized number of mapped reads values using quantile–quantile plots and determined that the distributions followed a theoretical normal distribution. We then used the ‘var.test’ function in R and determined that there was no significant difference in the variance in the coverage or in the number of mapped reads for *bla*_CTX-M-8_+ and *bla*_CTX-M-8_- genomes when mapped to *bla*_CTX-M-8_+ or *bla*_CTX-M-8_- plasmids. Following these results, we performed parametric two-sample *t*-tests with false discovery rate (FDR) correction to compare the means of coverage and normalized number of mapped reads between *bla*_CTX-M-8_+ and *bla*_CTX-M-8_- genomes when mapped to *bla*_CTX-M-8_+ and *bla*_CTX-M-8_- plasmids, respectively (FDR-adjusted *P* values <0.01 were considered statistically significant). In addition, we also conducted this analysis separately for each of the 30 reference plasmids (20 *bla*_CTX-M-8_+ and 10 *bla*_CTX-M-8_-; Dataset S15).

### Association between insertion sequence elements and *bla*_CTX-M-8_

All ST548 genomes were screened for insertion sequence (IS) elements using ISEScan v1.7.3 (default parameters). Phi correlation coefficients were measured between AMR gene presence/absence and IS element presence/absence using the ‘cor’ function from the stats package v4.3.2 in R (Dataset S16).

### Identification of *bla*_CTX-M–8_+ and other ZA ST548 genomes in the AllTheBacteria dataset

To assess the prevalence of *bla*_CTX-M-8_ in other bacterial lineages (e.g. other species, other *Salmonella* serotypes and sequence types and other ST548 genomes sequenced after our Enterobase data freeze), AMRFinderPlus results from AllTheBacteria (ATB) v0.2 (https://allthebacteria.readthedocs.io/en/latest/amr.html) were downloaded [63]. The ATB AMRFinderPlus results were then searched for ‘blaCTX-M-8’ (354 distinct genomes, Dataset S17). All *bla*_CTX-M-8_-harbouring ATB genomes classified as ‘*Salmonella enterica*’ were downloaded (*n*=64), and the resulting genomes underwent *in silico* seven-gene MLST using mlst v2.9 with default parameters. Sixteen genomes were assigned to ST548, and 5 of these genomes were already present in our full ST548 dataset, leaving us with 11 new genomes. These 11 genomes, plus the 23 existing *bla*_CTX-M-8_+ genomes (see the ‘Analysis of *bla*_CTX-M-8_-harbouring contigs’ section), underwent all-vs-all pairwise SNP distance estimation as described above (see the ‘Core SNP calling and phylogeny construction’ section; Dataset S18). Metadata for all 354 *bla*_CTX-M-8_-harbouring genomes from ATB were extracted using Entrez Direct v22.4 (Dataset S19) [64].

## Results

### Multiple *S*. Minnesota ST548 lineages can be detected in South African poultry imports of Brazilian origin

A total of 36 *S. enterica* strains isolated from frozen raw poultry samples collected between 2020 and 2022 underwent WGS (referred to as ‘SEPI’ genomes; see the ‘Sample isolation, DNA extraction, and WGS’ section). While all 36 strains were isolated within South African borders, the frozen raw poultry from which each strain was isolated originated from Brazil. More specifically, (i) 11 out of 36 genomes (31%) were isolates originating from Brazilian poultry meat collected for regulatory compliance, at POE; (ii) the remaining 25 isolates (69%) were from imported Brazilian poultry meat placed for sale in South African supermarkets (referred to hereafter as ‘POE’ and ‘supermarket’ genomes, respectively, Table S1).

Using *in silico* seven-gene MLST, all SEPI genomes belonged to ST548. *In silico* serotyping identified 35 genomes as serotype Minnesota (antigenic formula 21:b:e,n,x) by both SISTR and SeqSero2. One genome (strain 73, NCBI BioSample accession SAMN47166240) was assigned a serotype of ‘4:r:e,n,x’ by SeqSero2 and ‘-:b:e,n,x’ by SISTR. However, using core genome MLST (via SISTR), all SEPI genomes were identified as serotype Minnesota and will thus be referred to hereafter as *S*. Minnesota (see the ‘*In silico* serotyping and sequence typing’ section).

**Fig. 1.**
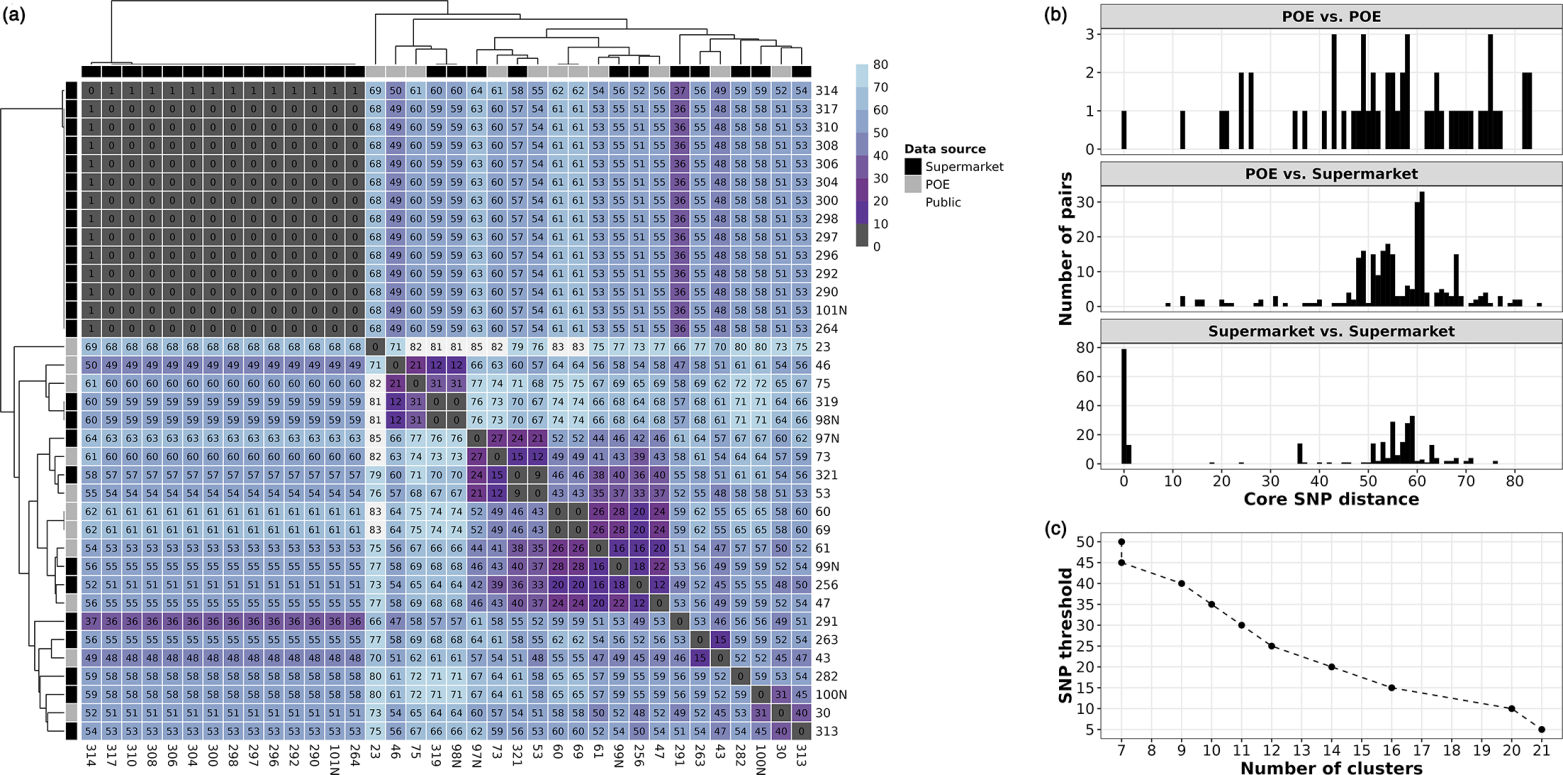
Core genome SNP distances show that multiple *S.* Minnesota ST548 lineages can be detected in Brazilian poultry imported into South Africa. (a) Heatmap showing pairwise core SNP distances calculated between the 36 ST548 genomes sequenced here (i.e. SEPI genomes). For the heatmap cells, darker shading indicates lower core SNP distances (i.e. higher sequence identity), while lighter shading indicates higher core SNP distances. Dendrograms were produced via average linkage hierarchical clustering of core SNP distances. Colour strips above and to the left of the heatmap denote the source of isolation for each genome (‘Data source’). POE, port-of-entry; Supermarket, imported poultry from supermarkets in South Africa. (b) Histogram of pairwise core SNP distances among the SEPI genomes separated by a specific source of isolation. ‘POE vs. POE’, comparisons within port-of-entry genomes only; ‘POE vs. Supermarket’, comparisons between port-of-entry and supermarket genomes; ‘Supermarket vs. Supermarket’, comparisons within supermarket genomes only. (c) Core SNP distance-based clustering of SEPI genomes. The *X*-axis shows the number of clusters at the corresponding core SNP threshold in the *Y*-axis. Clusters were obtained via average linkage hierarchical clustering (see the ‘Core SNP calling and phylogeny construction’ section).

Despite belonging to the same ST and despite sharing a similar origin (i.e. frozen raw poultry imported into South Africa from Brazil), considerable genomic diversity could be observed across SEPI genomes. SEPI genomes differed by 0–85 core SNPs after excluding SNPs likely due to recombination (mean 46, median 55; Fig. 1a). The range of core SNP distances was not affected by the specific source of each genome, i.e. the core SNP distance range was ~0–85, even when comparing supermarket genomes only or POE genomes only (Fig. 1b). Using average linkage hierarchical clustering of the pairwise core SNP distances, SEPI genomes could be partitioned into 7 SNP clusters at a threshold of 50 SNPs and to 21 SNP clusters at a threshold of 5 SNPs (Fig. 1c). Altogether, these results indicate that multiple *S*. Minnesota ST548 lineages are present in our collection of isolates from Brazilian frozen poultry imports.

### *S*. Minnesota ST548 strains detected in South African supermarkets are closely related to strains isolated from Brazilian poultry imported into the UK

To gain insight into the evolution of South African ST548 within the context of the global ST548 population, we compared our 36 SEPI genomes to 460 high-quality, publicly available ST548 genomes (*n*=496 total ST548 genomes; see the ‘Acquisition of publicly available WGS data and metadata’ section). Using Snippy, a total of 6,422 core SNP sites were identified among all 496 ST548 genomes, with SNP distances ranging from 0 to 346 (mean 160.5, median 193; Fig. S2A).

For each SEPI genome sequenced here, we identified the corresponding publicly available genome with the lowest SNP distance. This approach identified 25 distinct publicly available genomes (ties were kept), which differed from SEPI genomes by 1–26 core SNPs (Table S2). Notably, 18 SEPI genomes (17 supermarket genomes) were 19–27 SNPs apart from the same Brazilian poultry-associated public genome (NCBI Assembly accession GCA_006209225.1). This suggests that nearly 70% of our supermarket genomes (17 out of 25 genomes) may share a recent common ancestor with a poultry-associated isolate from Brazil. In addition, 11 other ST548 strains from a Public Health England collection (NCBI BioProject accession PRJNA248792) were 5–50 core SNPs apart from all SEPI genomes. All 11 of these strains, though isolated in the UK, were associated with Brazilian imported poultry [4]. This links SEPI isolates from South Africa with previously sequenced, poultry-associated strains from the UK, all of which share a common Brazilian origin.

### *S*. Minnesota ST548 strains from South African poultry imports of Brazilian origin are confined to a largely AMR clade

**Fig. 2.**
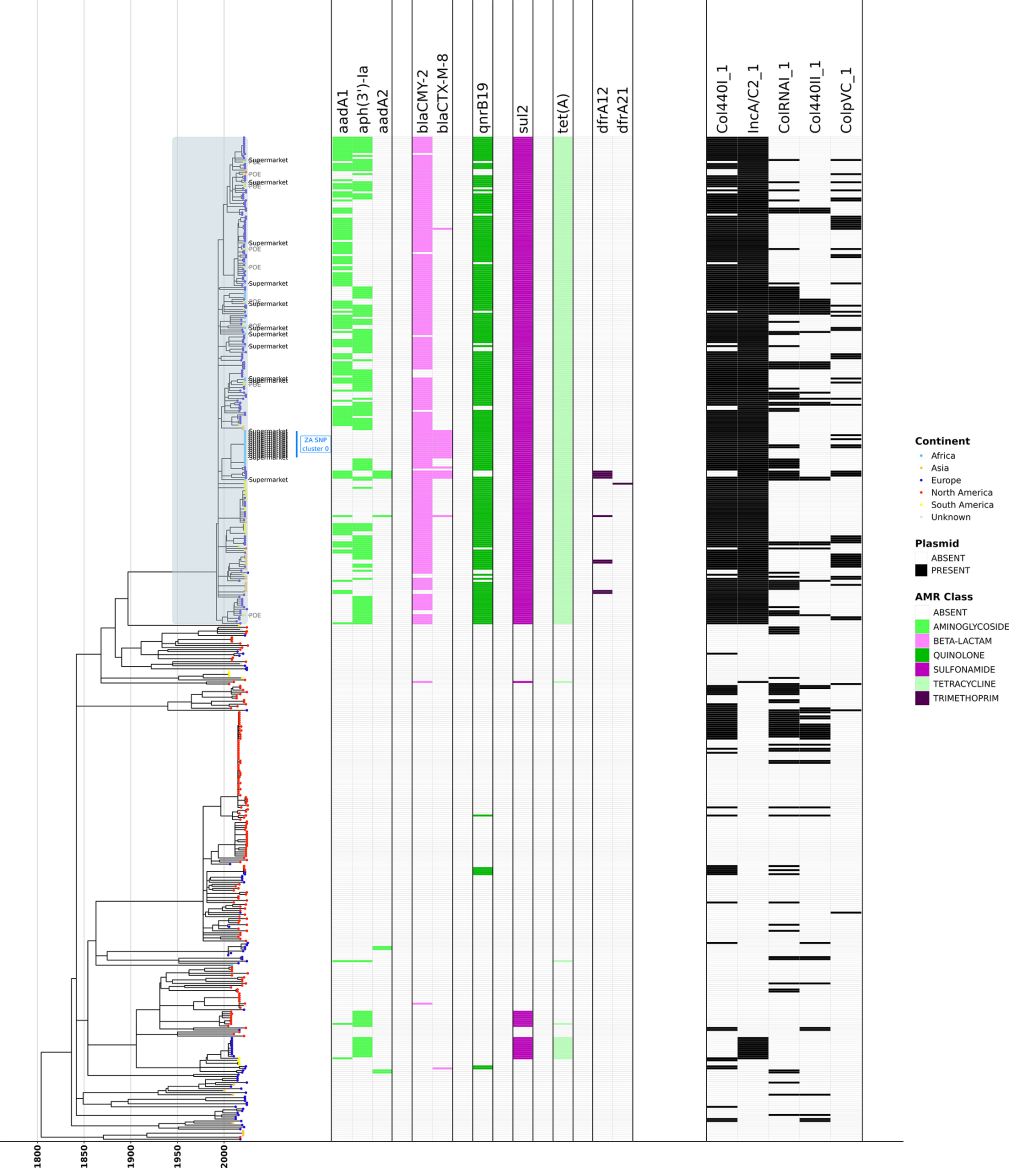
LSD2-estimated-rate ML phylogeny of *S.* Minnesota ST548 shows that the majority of AMR genes are confined to a single clade (*n* = 496; 36 SEPI genomes, plus 460 high-quality, publicly available *S.* Minnesota ST548 genomes). The ‘high-AMR group’ is denoted in the phylogeny by light blue shading. Tree tips for the 36 SEPI genomes are annotated with the isolation source (‘POE’ for strains isolated at the port of entry; ‘Supermarket’ for poultry samples from supermarkets in South Africa). Unlabelled tips denote publicly available genomes from Enterobase. Tips are coloured by continent of isolation (‘POE’ and ‘Supermarket’ genomes sequenced in this study were classified as ‘South American’ and ‘African’ isolates, respectively). Heatmaps to the right of the phylogeny denote (from left to right) (i) the presence/absence of AMR genes detected by AMRFinderPlus, coloured by AMR class, and (ii) the presence/absence of plasmid replicons (detected using ABRicate/PlasmidFinder). For readability, only selected AMR genes and plasmid replicons are shown (see Fig. S5 for the full version of this figure). The ML phylogeny was constructed using IQ-TREE, and the evolutionary rate was estimated by LSD2, with branch lengths reported in years (to view phylogenies constructed using different rooting/scaling methods, see Figs S3 and S4).

An ML phylogeny constructed using the 6,422 core SNPs detected among all 496 ST548 genomes revealed a clear separation in line with geographical origin, AMR profile and core SNP distances (Figs 2 and S2A, B). The LSD2-estimated-rate phylogeny showed an evolutionary rate of 1.16587e−07 substitutions/site/year, confidence interval (8.51142e−08; 1.448e−07) and time to most recent common ancestor (tMRCA) of 1803.56 (1723.1; 1849.547), and the TempEst estimated rate was 9.24e−07 substitutions/site/year with a tMRCA of 1967 (see the ‘Core SNP calling and ML phylogeny construction for the full ST548 dataset’ section). One clade comprising 241 genomes of mainly European, African, Asian and South American origin (ultrafast bootstrap support=99%; referred to hereafter as the ‘high-AMR group’) was found to contain 91% of all AMR genes detected in our total ST548 sample set (1,295 of 1,408 distinct AMR genes; Figs 2, S3, S4 and S5). Further, all 240 out of 241 genomes in the high-AMR group were predicted to be multidrug-resistant (MDR), i.e. harboured AMR genes conferring resistance to ≥3 antibiotic classes. The high-AMR group harboured 68% (149 of 218) of all European, 97% (37 of 38) of all African, 76% of all Asian (22 out of 29) and 77% (31 of 40) of all South American ST548 genomes (Figs S5 and S6). Only 2 out of 168 North American genomes were found in the high-AMR group. Importantly, all 36 of our SEPI genomes were found in the high-AMR group. The remaining genomes from Europe, Africa, Asia and South America (*n*=86), along with 99% of North American (*n*=166) genomes, belonged to other clades within the ST548 phylogeny and contained only ~8% of all AMR genes detected (113 of 1,408 distinct AMR genes; *n*=154 total genomes, referred to hereafter as the ‘low-AMR group’). A predicted MDR phenotype was observed among low-AMR group genomes only sporadically (22 of 255 low-AMR group genomes, 8.6%; Fig. S5). The evolutionary rate for the 241 genomes in the high-AMR group was estimated by LSD2 to be 4.91751e−07 substitutions/site/year [confidence intervals (4.1705e−07; 5.62379e−07)], with a tMRCA of 2008.77 [confidence intervals (2005.38; 2011.2); see the ‘Core SNP calling and ML phylogeny construction for the high AMR group’ section].

**Fig. 3.**
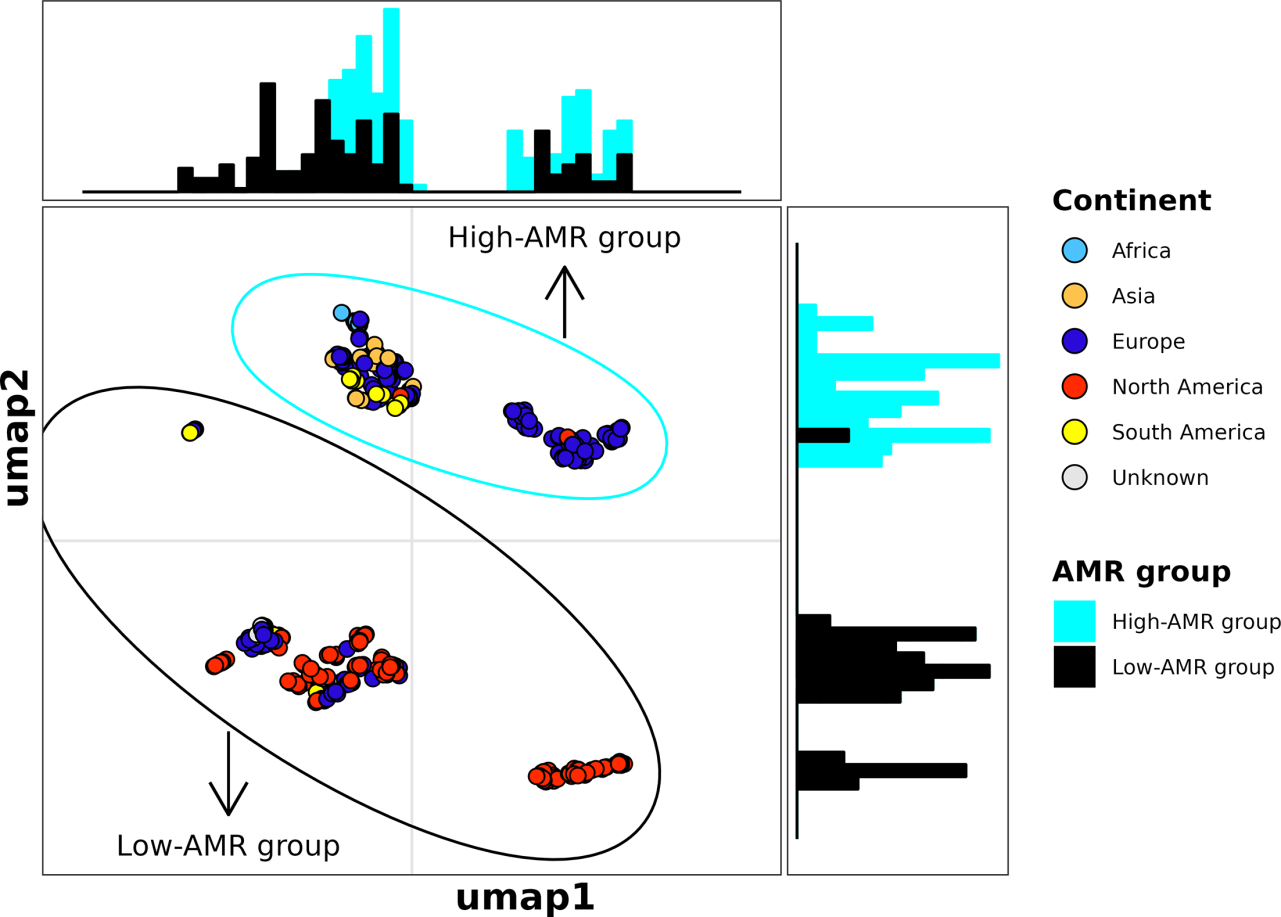
UMAP constructed using accessory gene presence/absence reveals separation between high-AMR and low-AMR groups. The UMAP was constructed from a presence/absence matrix of intermediate genes detected among 496 *S.* Minnesota ST548 genomes (i.e. genes present in >5% and <95% of the population according to Panaroo). Each point represents a genome, coloured by the reported continent of isolation. Ellipses encompass all genomes from the high-AMR group (cyan) or low-AMR group (black). Histograms along the *X-* and *Y-*axes of the plot represent the distribution of high-AMR group and low-AMR group genomes along the corresponding axis in the UMAP.

The separation between the high- and low-AMR groups was also evident when ST548 genomes were clustered by accessory gene presence/absence (i.e. genes present in >5% and <95% of ST548 per Panaroo). As was the case with the core genome ML phylogeny, all SEPI genomes clustered with publicly available genomes from the high-AMR group (Fig. 3). Interestingly, two genomes from the low-AMR group clustered near high-AMR group genomes (NCBI Assembly accessions GCA_011578495.1 and GCA_022318025.1; Fig. 3). These two genomes belonged to the small group of sporadic AMR+isolates in the low-AMR group and were positive for aminoglycoside, sulphonamide and tetracycline resistance genes (Fig. S5).

### Antimicrobial resistance genes in *S*. Minnesota ST548 are largely plasmid-associated

**Fig. 4.**
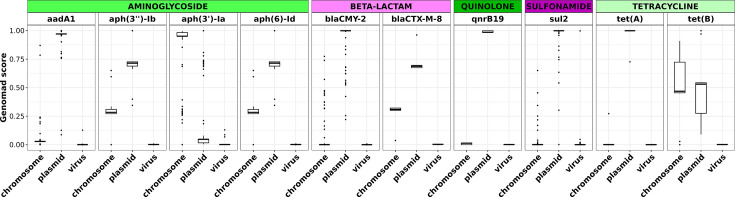
geNomad scores for ST548 AMR+ contigs show that most AMR genes are plasmid-associated. Boxplots showing geNomad calibrated aggregated classification scores for AMR gene-harbouring (AMR+) contigs present among 496 *S.* Minnesota ST548 genomes. Black horizontal lines within each boxplot denote the median geNomad score, and boxes show the interquartile range. Whiskers represent values up to 1.5× the first (lower) or third (higher) quartile, with black points showing outliers beyond the whiskers’ range. Each facet shows the geNomad chromosome, plasmid and phage score for the corresponding AMR gene. AMR genes are grouped by AMR class.

To investigate whether AMR genes from the high-AMR group were plasmid-associated, geNomad was used to predict the origin (chromosomal, plasmid or viral) of assembled contigs in the full ST548 set (Fig. 4). geNomad predicted plasmid scores for contigs harbouring AMR genes (AMR+ contigs) were then compared to contigs without AMR genes (AMR- contigs) as detected by AMRFinderPlus. Overall, plasmid scores were significantly greater for AMR+ contigs compared to AMR- contigs (*P*<2.22e−16, Mann–Whitney *U* test; Fig. S7).

To investigate the distribution of AMR genes on plasmids, we linked all ≥1,000 bp contigs annotated with AMR genes to those mapping to plasmids in PLSDB with >95% coverage and >95% identity by blastn (see the ‘Identification of plasmid-mediated AMR genes’ section). We then compiled, for each genome, the set of AMR genes that co-occurred on the same plasmid-associated contig(s) and filtered for MDR-conferring plasmids (i.e. plasmids harbouring AMR genes conferring resistance to ≥3 antibiotic classes). The most frequent combination was aminoglycoside+beta-lactam+tetracycline, co-occurring on the same plasmid in 161 genomes. Beta-lactam+tetracycline+sulphonamide was the second most frequent combination, co-occurring on the same plasmid in 90 different genomes. In addition, >85% of the candidate plasmids harbouring one of the above 2 AMR combinations were >100,000 bp in length. Important caveats to this analysis are that we cannot pinpoint the exact plasmid(s) present, as there are multiple potential matching reference plasmids. Additionally, we cannot resolve the exact plasmid-AMR combination in each genome due to the fragmented nature of short-read assemblies. However, the AMR-contig and plasmid-contig co-occurrences provide evidence that MDR-conferring megaplasmids are in circulation in this lineage, as has also been noted previously [16].

### A bla_CTX-M-8_-harbouring plasmid from *E. coli* has entered South Africa via Brazilian poultry imports

Among the 36 SEPI genomes sequenced here, the largest SNP cluster consisted of 14 isolates, all 0 core SNPs apart (referred to hereafter as ‘ZA SNP cluster 0’). All 14 ZA SNP cluster 0 genomes were isolated from poultry meat sold in supermarkets in South Africa (Figs1a  2). A unique feature of genomes within the ZA SNP cluster 0 was that they harboured *bla*_CTX-M-8_, which was otherwise absent in all other ZA genomes in our dataset. This was in addition to *bla*_CMY-2_, *sul2* and *tet*(A), which were, as stated previously, potentially carried by a megaplasmid (see the ‘Antimicrobial resistance genes in *S*. Minnesota ST548 are largely plasmid associated’ section). We also found *bla*_CTX-M-8_ in 8 ST548 genomes from the UK isolated between 2022 and 2023.

To assess the prevalence of *bla*_CTX-M-8_ in other *S. enterica* lineages, we searched a catalogue of AMR determinants detected in >1.9 million bacterial genomes (i.e. AMRFinderPlus results from the ATB dataset) [63]. Out of 644,699 ATB *Salmonella enterica* genomes, only 64 possessed *bla*_CTX-M-8_ (0.01%), suggesting that *bla*_CTX-M-8_ is not common in *Salmonella enterica* (see ‘Identification of *bla*_CTX-M–8_+ and other ZA ST548 genomes in the AllTheBacteria dataset’ in the Methods above). Out of these 64 *bla*_CTX-M-8_-harbouring (*bla*_CTX-M-8_+) ATB *Salmonella enterica* genomes, 16 belonged to ST548 (25%), five of which were already included in our full ST548 dataset. The remaining 11 *bla*_CTX-M-8_+ ST548 ATB genomes were not included in our initial dataset because there was no associated Enterobase record at the time of our data freeze, or the existing assembly did not pass our quality filters (see the ‘Acquisition of publicly available WGS data and metadata’ section). Further investigation of all 34 *bla*_CTX-M-8_+ genomes (14 ZA SNP cluster 0+8 Enterobase +12 ATB) revealed that the public genomes differed from ZA SNP cluster 0 by 11–317 core SNPs (Fig. S8). The two public genomes that differed from ZA SNP cluster 0 by >250 core SNPs were not part of the high-AMR group itself (Figs S2B, S8). Interestingly, one of the 12 *bla*_CTX-M-8_+ ST548 ATB genomes was reported to be from South Africa (NCBI BioSample accession SAMEA14452848), isolated in 2022 from animal meat along with ten other *bla*_CTX-M-8_- samples. Each of these 11 South African samples was 9–42 core SNPs apart from at least one SEPI genome sequenced here (Table S3). From the remaining 15 *bla*_CTX-M-8_+ ST548 genomes, five were from Brazilian poultry, isolated between 2016 and 2022; nine were reported to be isolated from ‘food’ in Portugal and the UK. The one remaining sample from the UK was reported to be isolated from a human (NCBI BioSample accession SAMN30444884). Taken together, our results suggest that while *bla*_CTX-M-8_ is relatively rare in *Salmonella enterica*, ST548 has been subjected to multiple independent introductions of *bla*_CTX-M-8_.

**Fig. 5.**
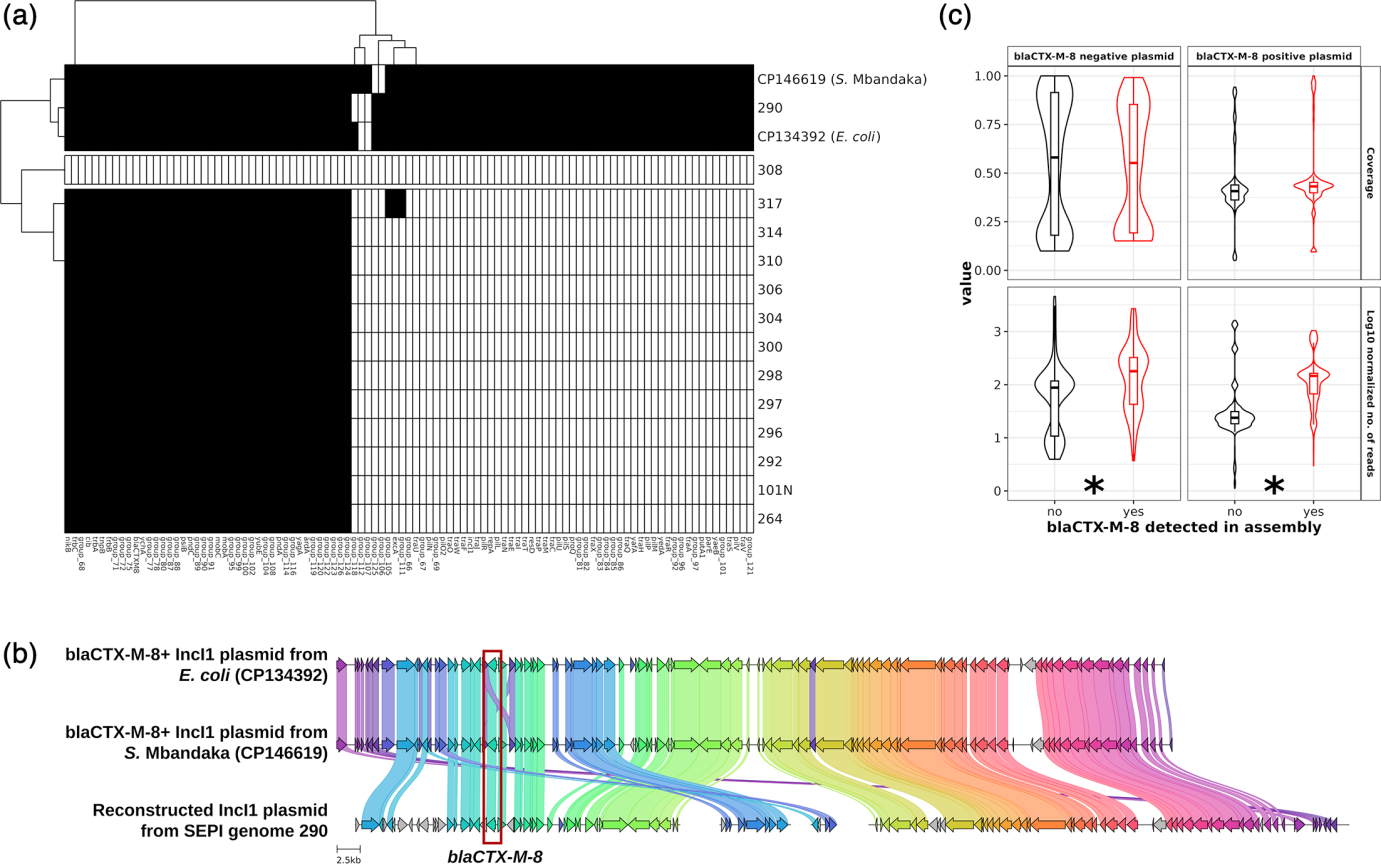
*bla*_CTX-M-8_-harbouring contigs from ZA SNP cluster 0 are highly similar to an IncI1 plasmid from *S.* Mbandaka and *E. coli*. (a) Heatmap showing pangenome gene presence/absence in plasmids reconstructed from SEPI genomes when compared to *bla*_CTX-M-8_-harbouring reference plasmids CP146619.1 (*S.* Mbandaka) and CP134355.1 (*E. coli*). Black cells indicate presence and white cells indicate absence. Gene cluster labels according to Panaroo are shown in the *x*-axis and plasmid names are shown in the *y*-axis. Plasmids reconstructed from the SEPI genomes are named after the SEPI genomes themselves. (b) Plot showing the synteny and sequence identity of *bla*_CTX-M-8_-harbouring IncI1 plasmids detected in SEPI genome 290 and reference plasmids detected in *S.* Mbandaka (CP146619.1) and *E. coli* (CP134355.1). The bands connecting each gene (arrows) indicate amino acid sequence identity, with colours indicating gene cluster groups. Sequences with <95% identity are not connected. (c) Violin plots showing coverage (top) and log10-transformed *rpoD*-normalized number of mapped reads (bottom). Reads from 171 ST548 genomes were mapped to 10 *bla*_CTX-M-8_- and 20 *bla*_CTX-M-8_+ plasmids from PLSDB (see the ‘Read mapping against *bla*_CTX-M-8_+ plasmids’ section). The *x*-axis shows genomic *bla*_CTX-M-8_ status, and the facets show plasmid *bla*_CTX-M-8_ status. The ‘*’ indicates a statistically significant difference (t.test with FDR-adjusted *P*<0.01) between the *bla*_CTX-M-8_+ and *bla*_CTX-M-8_- genome groups, and absence of ‘*’ indicates no statistically significant difference.

A IncI1 pST113 *bla*_CTX-M-8_+ plasmid has previously been reported in *S. enterica* serotype Mbandaka (*S*. Mbandaka; NCBI Nucleotide accession CP146619.1), where it was also hypothesised that the plasmid may be acquired from *E. coli* [60]. Based on this, we initially sought to test if *bla*_CTX-M-8_ was carried by the same plasmid in ST548. All *bla*_CTX-M-8_+ contigs from ZA SNP cluster 0 matched both the *S. Mbandaka* and an IncI pST113 reference plasmid from *E. coli* (NCBI Nucleotide accession CP134355.1) with >95% coverage and >95% identity. In an attempt to assemble the complete plasmid, we ran plasmidSPAdes on the trimmed reads from each ZA SNP cluster 0 genome. We then annotated the contigs from each plasmid assembly with Bakta and compared the resulting gene clusters with Panaroo. We found that only one ZA SNP cluster 0 genome (genome 290)-associated plasmid assembly had >95% of the genes found in the two reference plasmids (CP146619.1 and CP134355.1), with the remaining 13 ZA SNP cluster 0 genomes having <50% (Fig. 5a). The gene cluster alignments from the reconstructed SEPI genome 290 *bla*_CTX-M-8_+ plasmid with the *S*. Mbandaka and *E. coli bla*_CTX-M-8_+ IncI plasmids showed consistent synteny; however, due to the use of short-read data, the plasmid reconstruction remained fragmented (Fig. 5b). While our PlasmidFinder results did not show the presence of IncI1 in any of the ZA SNP cluster 0 genome assemblies (Fig. S3, see the ‘AMR determinant and plasmid replicon detection’ section), IncI1 was detected in the plasmidSPAdes assembly for genome 290 (see the ‘Reconstruction of blaCTX-M-8 + plasmid from ZA SNP cluster 0 genomes’ section). This may be because the genome assembler used here (SEKSA) was unable to resolve low coverage/low depth regions that a plasmid-aware assembler (plasmidSPAdes) retained.

To investigate further, we independently mapped reads from all 36 SEPI genomes (14 *bla*_CTX-M-8_+ ZA SNP cluster 0 genomes, plus 22 *bla*_CTX-M-8_- genomes) to both the *E. coli* and *S. Mbandaka* IncI1-pST113 plasmids, as well as all other *bla*_CTX-M-8_+ plasmids in a non-redundant version of PLSDB (*n*=21; 17 IncI1 plasmids, 2 IncL/M(pMU407), 1 IncN and 1 IncX1). We also performed read mapping against reference plasmids that do not harbour *bla*_CTX-M-8_ (*n*=10; see the ‘Identification of plasmid-mediated AMR genes’ and ‘Read mapping against *bla*_CTX-M-8_+ plasmids’ sections). Though we found on average more reads from *bla*_CTX-M-8_+ genomes mapping to the reference plasmids, we consistently found only ~50% breadth of coverage regardless of *bla*_CTX-M-8_+ status (Fig. 5c). This suggests that while a plasmid backbone similar to IncI1-pST113 may be present in our SEPI genomes, it need not necessarily be a carrier of *bla*_CTX-M-8_ and that this backbone can circulate independently of *bla*_CTX-M-8_ . We also found that reads from genome 290 mapped with 100% coverage against the *E. coli* IncI1 reference plasmid (CP134355.1), further supporting the conclusion that the IncI1 operon is indeed present in genome 290 and that the region was not resolved during genome assembly.

We extended this analysis to the broader ST548 dataset, where we mapped reads from each genome in the SRA set (i.e. public genomes for which raw reads were downloaded; see the ‘Acquisition of publicly available data and metadata’ section), as well as the 12 *bla*_CTX-M-8_+ ST548 ATB genomes, to the 20 *bla*_CTX-M-8_+ plasmids and 10 *bla*_CTX-M-8_- plasmids from PLSDB (Fig. S9, see the ‘Read mapping against *bla*_CTX-M-8_+ plasmids’ section). We observed that while the coverage and number of mapped reads against each reference plasmid were significantly different between the *bla*_CTX-M-8_+ and *bla*_CTX-M-8_- genomes in some cases, these differences were independent of the plasmid *bla*_CTX-M-8_ status, as well as the genomic *bla*_CTX-M-8_ status. This further supports the conclusion that, in ST548, the *bla*_CTX-M-8_ allele is not always plasmid-mediated.

An IS6 family IS element (IS26) was reported to be found along with *bla*_CTX-M-8_ in the *S*. Mbandaka IncI pST113 plasmid [60]. We hypothesized that in cases where the complete pST113-like plasmid was not found, *bla*_CTX-M-8_ may have integrated into the chromosome through IS elements. In all ZA SNP cluster 0 genome assemblies, *bla*_CTX-M-8_ was found on a short (~2,100 bp) contig. Upon screening these contigs for IS elements, we found an IS element, IS4, in all cases (see the ‘Association between insertion sequence elements and *bla*_CTX-M-8_’ section). This suggests that *bla*_CTX-M-8_ may also be disseminated through an IS4 element.

We then screened our full ST548 dataset for IS elements and measured associations between IS element presence/absence and AMR gene presence/absence (see the ‘Association between insertion sequence elements and *bla*CTX-M-8’ section). While our results showed that IS4 and IS6 have strong associations with *bla*_CMY-2_ (Phi coefficient>0.75), we did not observe a strong correlation between *bla*_CTX-M-8_ and any IS element (Fig. S10). This suggests that while *bla*_CTX-M-8_ is associated with IS4 in our ZA SNP cluster 0 genomes, IS4 does not exclusively disseminate *bla*_CTX-M-8_, as we found IS4 to be strongly associated with *qnrB19*, *sul2* and *tet(A*) (Fig. S10).

Collectively, this showed that, in addition to an existing MDR-conferring megaplasmid commonly found in the high-AMR group of *S*. Minnesota ST548, 14 SEPI genomes have an additional mobile-element-associated *bla*_CTX-M-8_ gene. Using AMRFinderPlus results from ATB, we found a total of 354 genomes with *bla*_CTX-M-8_; of these, 248 were detected in *E. coli* (70.1%), including the earliest reported *bla*_CTX-M-8_+ sample in the ATB dataset (NCBI BioSample accession SAMN33422834, isolated in 2005 from a rectal swab of a dog in the United Arab Emirates; see the ‘Identification of *bla*_CTX-M–8_+ and other ST548 genomes in the AllTheBacteria dataset’ section). This suggests that the *bla*_CTX-M-8_+ IncI plasmid may have originally transferred to ST548 from *E. coli*, but the dissemination of *bla*_CTX-M-8_ may occur in a plasmid-independent manner. Due to the limitations of short-read assemblies, we are unable to conclusively resolve the genomic context of *bla*_CTX-M-8_ in all our genomes.

## Discussion

### POE genomic surveillance provides insight into the global dissemination of pathogens via agro-food trade

Bacterial pathogens endemic to a given geographical region can be exposed to unique, local selection pressures, which shape their evolution and population structure (e.g. exposure to antimicrobials in agricultural, environmental or clinical settings, dictated by local regulations or the lack thereof) [65,68]. For foodborne zoonotic pathogens like *S. enterica*, the global agro-food trade serves as a mechanism by which endemic pathogens can be disseminated to other world regions, potentially infecting humans and/or animals [4,5, 69,73]. As such, many countries routinely test agro-food imports for the presence of pathogens, with the goal of preventing contaminated imports from entering domestic food systems [73,75]. In South Africa, under the Meat Safety Act, 2000 (Act No. 40 of 2000) and related Veterinary Procedural Notice (VPN), microbiological testing is required for compliance monitoring of both domestic and imported meat products. Upon arrival and during storage, random samples are collected from selected consignment packages and sent to approved laboratories for bacteriological testing, including *Salmonella* detection [21]. Since 2018, VPN 56 mandates that all *Salmonella* strains isolated from meat samples in South Africa be forwarded to the ARC-OVR-General Bacteriology Laboratory for storage and molecular typing monitoring [76].

Here, we used WGS to characterize 36 *S*. Minnesota ST548 strains isolated in South Africa, from imported poultry of Brazilian origin. In addition to querying ST548 strains from poultry meat sold in supermarkets, our sampling efforts included ST548 strains isolated from poultry meat consignments held at POE. Our WGS data showed that *S*. Minnesota ST548 strains detected in South African supermarkets were closely related to strains isolated in poultry meat exported from Brazil to the UK. All 36 of our isolates were confined to a well-supported clade within the ST548 phylogeny (i.e. the ‘high-AMR group’), which contained at least 11 other isolates known to be associated with Brazilian poultry imports. These 11 isolates were reported in a previous study from the UK, in which *S*. Minnesota genomes from UK poultry meat imports of Brazilian origin formed a monophyletic clade with genomes from Brazil [4]. Further, 7 of our genomes were ≤10 core SNPs apart from genomes from the UK (Table S2). Based on a previously estimated ST548 evolutionary rate of 1.27e−7 [13] as well as our LSD2 estimated rate of 1.16e−7, SEPI genomes that are fewer than 10 core SNPs apart from the previously sequenced food-associated genomes indicate an evolutionary divergence period of less than a decade, suggesting recent shared ancestry. This indicated that some (but not all) European and African imports may share a common source. On the other hand, other SEPI genomes may represent more diverged lineages across longer timescales (>30 core SNPs apart or >3 decades of evolutionary divergence). We also found a set of 11 South African food-associated ST548 genomes, which were 9–42 core SNPs apart from at least one of our SEPI genomes (Table S3).

Overall, currently available WGS data suggest that several *S*. Minnesota ST548 lineages have been introduced into South Africa through Brazilian poultry imports. The results showcase the value of rich geographic metadata in pathogen surveillance studies, particularly metadata conveying the provenance of imported foods. As shown here and elsewhere [18,19, 21, 77, 78], foodborne pathogens isolated at POE, from a known exporter, are particularly useful, as they can capture pathogen migration events between geographic regions with known directionality.

### Existing publicly available WGS data cannot link *S*. Minnesota ST548 from Brazilian poultry imports to South African human clinical cases

Some importers have expressed concern regarding *Salmonella* in Brazilian meat and poultry [4,22, 79, 80]. However, the increasing prevalence of *S*. Minnesota in poultry may not necessarily translate to an increase in human salmonellosis cases. For example, in the UK, there was no observable increase in human clinical cases caused by *S*. Minnesota, despite the increasing prevalence of *S*. Minnesota in Brazilian poultry, and the few observed human clinical cases could largely be explained by recent international travel [4]. The European Centre for Disease Prevention and Control infectious disease surveillance atlas shows only 354 total reported cases of human salmonellosis caused by *S*. Minnesota from 2007 to 2023 in the EU (UK not included after 2019). Another common Brazilian poultry-associated serotype, *Salmonella* Heidelberg, showed 1,790 cases in the same time period, though with a decreasing trend. These figures are dwarfed by serotype *S*. Enteritidis, which showed 33,088 cases in the EU in 2023 alone (https://atlas.ecdc.europa.eu/public/index.aspx). Overall, these data suggest that *S*. Minnesota may not be a significant threat to human health compared to other *Salmonella* serotypes in Europe.

Here, we found that all *S*. Minnesota strains isolated in South Africa are <65 core SNPs apart from food/poultry-associated strains from Brazil or the UK (Fig. S2A, Tables S2 and S3). Therefore, it is possible that Brazilian *S*. Minnesota ST548 also does not impose a significant disease burden on South African consumers, as evidence indicates for the UK and EU [4]. However, a lack of links to clinical cases could rather be the result of data gaps. Despite imposing a disproportionately high burden of illness on sub-Saharan Africa [81,82], *Salmonella* WGS efforts are largely concentrated in world regions where the burden of salmonellosis is lower [81,83, 84]. For example, of the 496 Enterobase *Salmonella* ST548 genomes included in our dataset, 324 (~65%) were isolated in the US or the UK. While African-led pathogen surveillance efforts are producing an unprecedented amount of data, African WGS data generators can still face major hurdles when sharing, publishing and/or disseminating pathogen WGS (meta)data [85]. Thus, the lack of observed links between imported *S*. Minnesota and human clinical cases in South Africa could be due to the lack of publicly available WGS data from *S*. Minnesota strains in South Africa (e.g. from human clinical cases, as well as domestic animals, foods and environmental sources). Overall, the UK experience with *S*. Minnesota, both in terms of surveillance and disease burden, cannot be applied directly to South Africa, and our study highlights the importance of local pathogen surveillance initiatives, beyond those conducted in Europe and North America.

### Multiple *bla*_CTX-M-8_-harbouring *S*. Minnesota ST548 lineages have been disseminated via the international poultry trade

Antimicrobial use in livestock and food production environments can select for AMR foodborne zoonotic pathogens, which can be subsequently disseminated via international trade [86,89]. Consequently, farm management and antimicrobial use practices in a single country, region or even farm can impact human and animal health across the globe [89,90]. Here, we identified a cluster of genomes with high sequence identity (i.e. ZA SNP cluster 0, differing by 0 core SNPs), which had acquired *bla*_CTX-M-8_. *bla*_CTX-M-8_ was published as a novel CTX-M beta-lactamase in 2000 [91], when it was identified in three strains of three species (*Enterobacter cloacae*, *Enterobacter aerogenes* and *Citrobacter amalonaticus*) isolated in 1996–1997 from intensive care unit patients in Rio de Janeiro, Brazil. Since then, studies in Brazil and other South American countries have noted that *bla*_CTX-M-8_ appears to be relatively common among extended-spectrum beta-lactamase-producing *E. coli* in the region [92,93]. However, *bla*_CTX-M-8_ has been detected in numerous other countries, in a range of hosts (e.g. *E. coli*, *Shigella* spp., *Enterobacter* spp., *Klebsiella* spp. and *Salmonella enterica*), isolated not only from human clinical cases, but also from apparently healthy humans (e.g. working as food handlers), animals/animal products (e.g. chicken meat, chickens, cattle), wastewater and wildlife (e.g. gulls, wild birds) as well [92,104]. Most notably within the context of this study, *bla*_CTX-M-8_ has previously been detected in *E. coli* isolated from Brazilian poultry products imported into Japan [94,105], indicating that this AMR gene has previously been disseminated intercontinentally via Brazilian poultry.

In addition to the ZA SNP cluster 0, we identified an additional 20 *bla*_CTX-M-8_-harbouring *S*. Minnesota ST548 genomes, isolated between 2017 and 2022 (three samples had unreported isolation dates) from four different countries (i.e. Brazil, Portugal, South Africa and the UK; two samples had unreported isolation locations). Two of these *bla*_CTX-M-8_-harbouring *S*. Minnesota ST548 genomes (NCBI BioSample accessions SAMN30444884 and SAMEA12320398), one from Brazil and one from the UK, were >290 core SNPs apart from ZA SNP cluster 0 (Fig. S8). This indicates that multiple independent acquisition events are responsible for the emergence of *bla*_CTX-M-8_ in ST548. Previous studies have identified *bla*_CTX-M-8_ on plasmids, including IncI1 plasmids harboured by multiple *E. coli* STs [92,94, 102], indicating that multiple *E. coli* lineages have acquired this AMR gene via IncI1 plasmids. Further, a *bla*_CTX-M-8_-harbouring IncI1 plasmid was recently described in a *Salmonella* Mbandaka strain (isolated in 2022 from a broiler farm environment in Poland) [60]. The *bla*_CTX-M-8_ gene in this *S*. Mbandaka IncI1 plasmid was reported to be flanked by IS6 family elements. IS elements are a major mechanism driving chromosomal integration of AMR genes [106]. Previous studies have shown inter-species Inc1 plasmid mobility facilitating dissemination of other *bla*_CTX_ genes, with chromosomal integrations mediated by IS elements [107,108]. Here, we identified IS4 elements associated with all ZA SNP cluster 0 genomes, despite finding evidence of the complete IncI1 pST113 plasmid in only one case (Fig. 5; SEPI genome 290). This suggests both plasmid-dependent and plasmid-independent mechanisms of *bla*_CTX-M-8_ circulation have taken place within ST548. However, further validation using long-read sequencing would be required to confirm these results.

Overall, *bla*_CTX-M-8_ can be acquired within and across species boundaries via IncI1 plasmids and IS elements. Based on the genomic analyses conducted here, we hypothesize that ZA SNP cluster 0 acquired *bla*_CTX-M-8_ recently, within Brazil, via an *E. coli* IncI1 plasmid (or associated IS elements), and was subsequently disseminated to South Africa via imported poultry. However, whether *bla*_CTX-M-8_-harbouring *S*. Minnesota imposes a significant burden on humans and animals in South Africa remains a mystery. Future WGS efforts targeting *S*. Minnesota from human and non-human sources (e.g. poultry flocks, farm environments and poultry products) in South Africa are needed. We anticipate that long-read sequencing methods, in particular, will provide insight into plasmid-mediated AMR dynamics within *S*. Minnesota. This, combined with continued surveillance of imported foods at port of entry, will allow for improved risk evaluation of imported foods in South Africa.

## Supplementary material

10.1099/mgen.0.001633 Uncited Supplementary Material 1.

10.1099/mgen.0.001633 Uncited Supplementary Material 2.

## Abbreviations

AMR: antimicrobial resistance
ARC-OVR: Agricultural Research Council–Onderstepoort Veterinary Research
ATB: AllTheBacteria
FDR: false discovery rate
IS: insertion sequence
MDR: multidrug-resistant
ML: maximum likelihood
MLST: multi-locus sequence typing
NCBI: National Center for Biotechnology Information
POEs: ports of entry
SEPI: *Salmonella enterica* Poultry Imports
SRA: Sequence Read Archive
ST548: sequence type 548
tMRCA: time to most recent common ancestor
UMAP: Uniform Manifold Approximation and Projection for Dimension Reduction
VPN: Veterinary Procedural Notice
WGS: whole-genome sequencing
