# Supplementary material for: Genomic surveillance of Salmonella enterica serotype Minnesota strains from poultry products imported into South Africa
Source: Microb Genom. 2026 Feb 5;12(2):001633. doi: 10.1099/mgen.0.001633 (PMC12877407; doi:10.1099/mgen.0.001633)
Supplement: Uncited Supplementary Material 1. [file mgen-12-01633-s001.pdf]

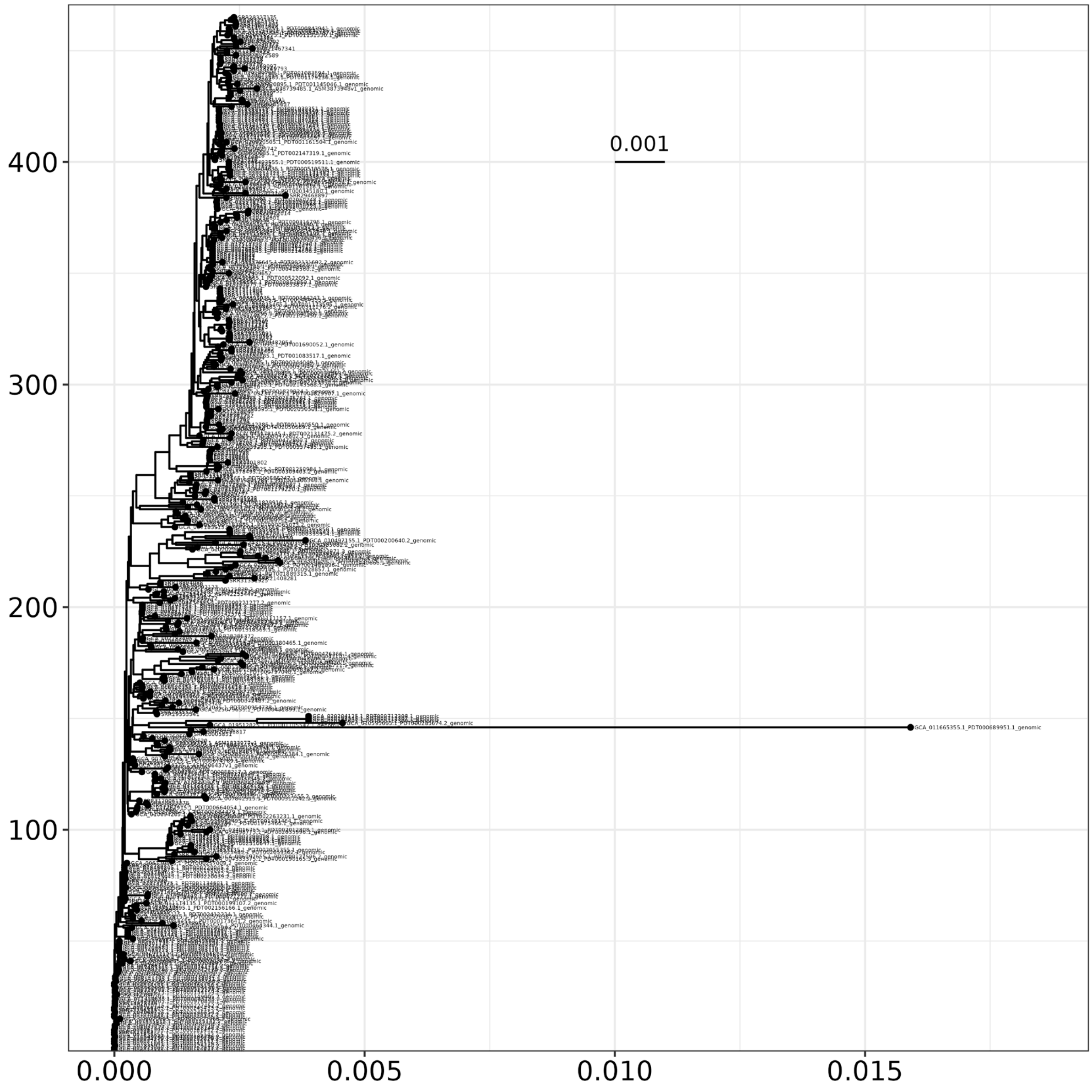

Fig S1: Mash distance-based tree of 465 curated publicly available *S*. enterica ST548 genomes revealed a single outlier sample (GCA_011665355.1) which was then removed from the dataset before further analyses.

High quality versions of all figures are available in https://github.com/VishnuRaghuram94/SEPI/tree/main/manuscript_figs/png

Open Rubric

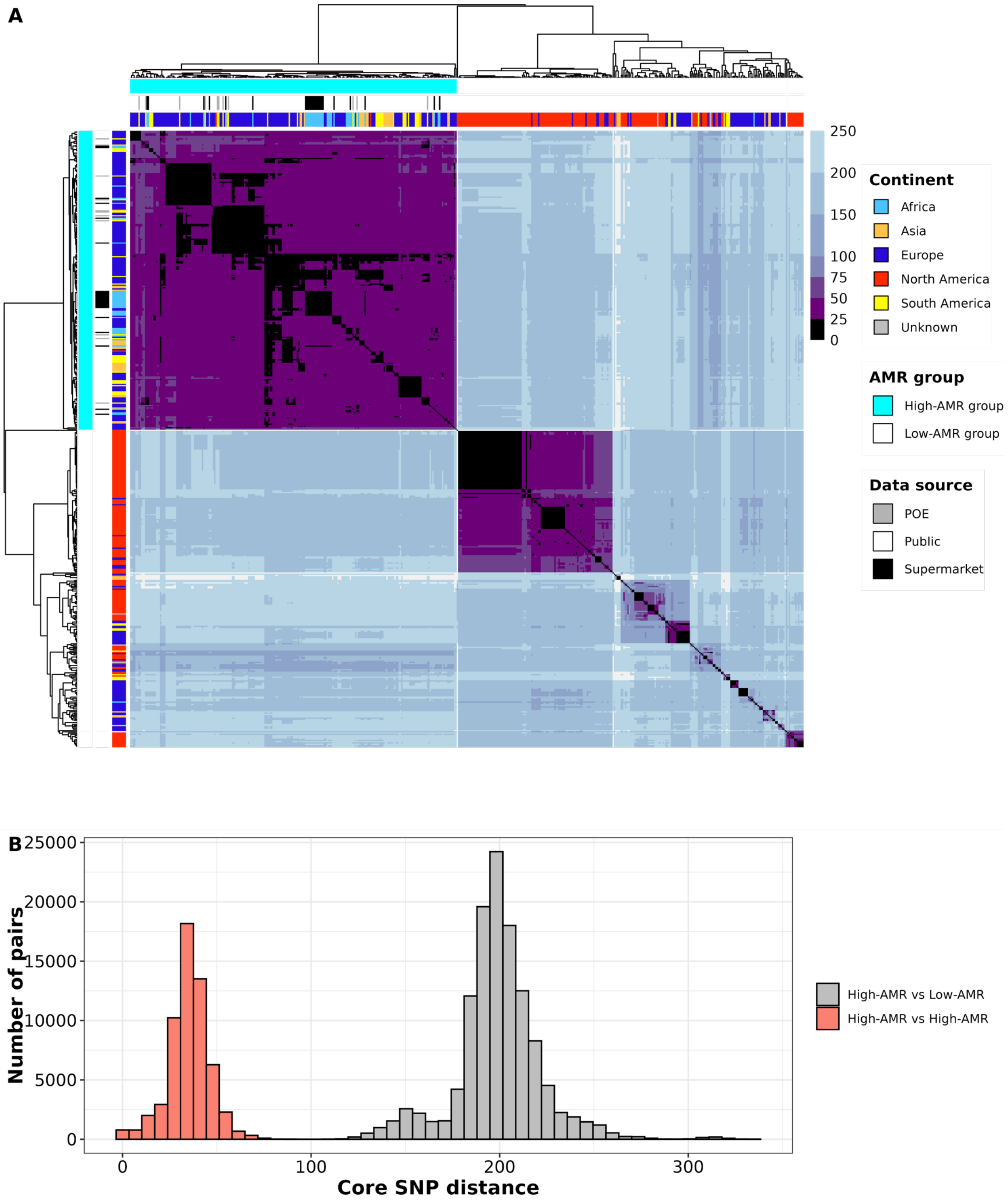

Fig S2: (A) Heatmap showing pairwise core genome SNP distance (lighter = higher SNP distance) for all 496 ST548 isolates. Heatmap is annotated by 1) continent of isolation; 2) source of the data (POE - Port of entry, Supermarket - poultry samples from supermarkets in South Africa, Public data - ST548 genomes uploaded to Enterobase) and 3) AMR prevalence (high-AMR group/low-AMR group). **(B)** Histogram of pairwise core SNP distances between only genomes within the high-AMR group (red bars) or between the high-AMR group and low-AMR group (grey bars).

High quality versions of all figures are available in https://github.com/VishnuRaghuram94/SEPI/tree/main/manuscript_figs/png

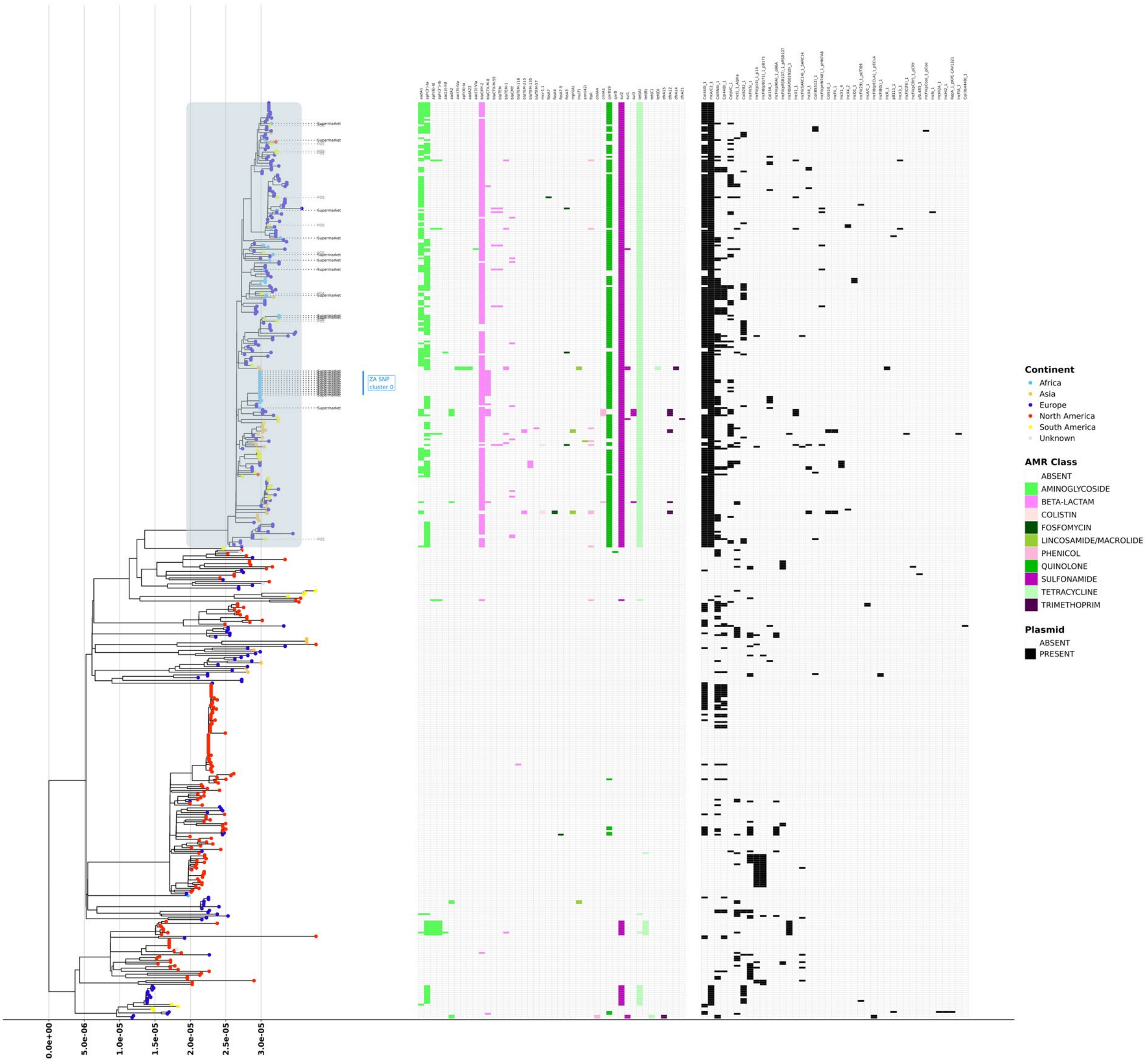

Fig S3: Midpoint rooted maximum-likelihood (ML) phylogenetic tree constructed using IQ-TREE comprising all 496 ST548 isolates.Tree tips are coloured by continent of isolation. Heatmap corresponding to tree tips indicate the source of the data (POE - Port of entry, Supermarket - poultry samples from supermarkets in South Africa, Public data - ST548 genomes uploaded to Enterobase) and presence/absence of antimicrobial resistance (AMR) genes as detected by AMRFinderPlus coloured by AMR class, and presence/absence of plasmid replicons as detected by Abricate.

High quality versions of all figures are available in https://github.com/VishnuRaghuram94/SEPI/tree/main/manuscript_figs/png

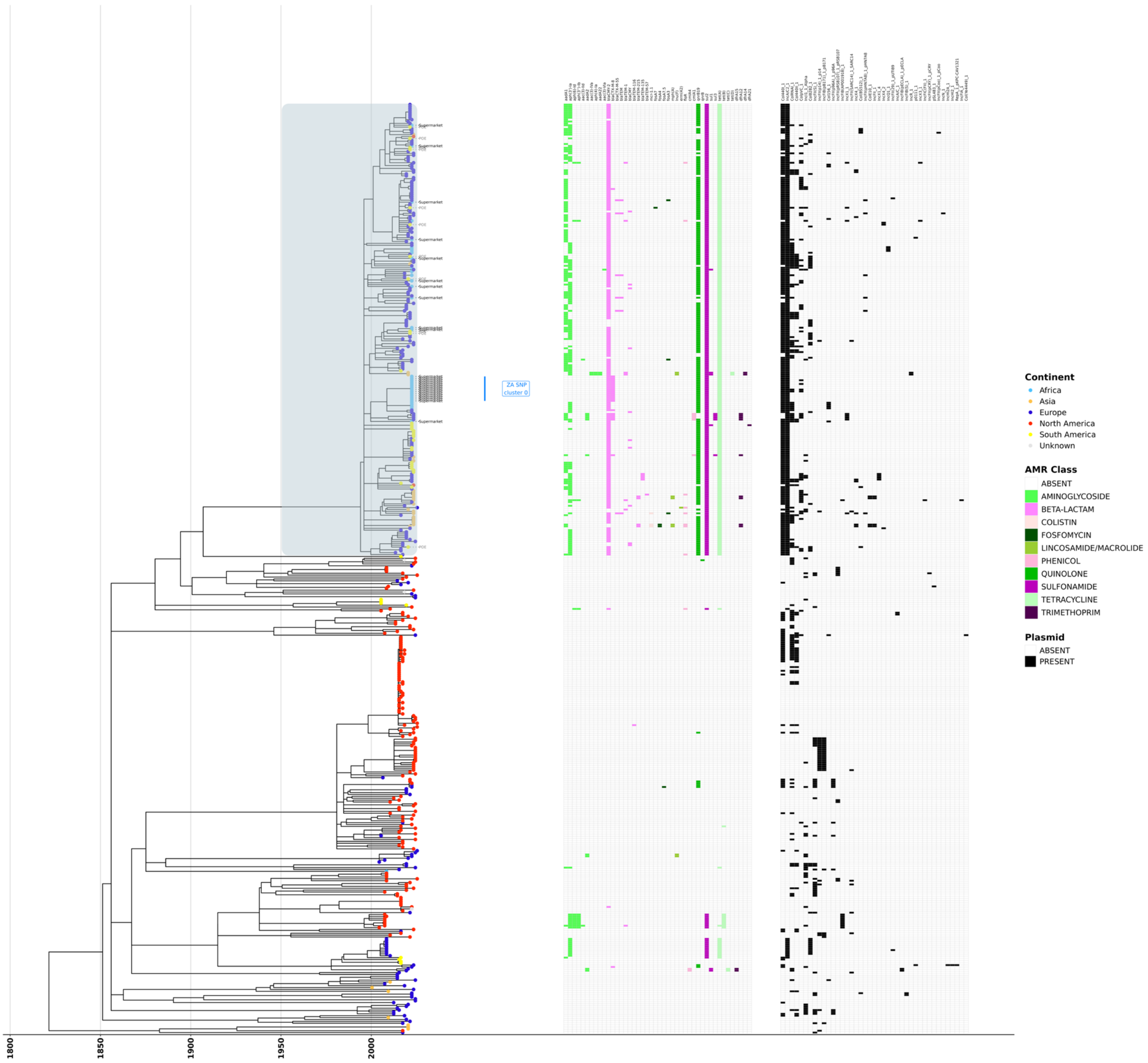

Fig S4: Fixed-rate maximum-likelihood (ML) phylogenetic tree constructed using IQ-TREE and RLSD2 comprising all 496 ST548 isolates.Tree tips are coloured by continent of isolation. Heatmap corresponding to tree tips indicate the source of the data (POE - Port of entry, Supermarket - poultry samples from supermarkets in South Africa, Public data - ST548 genomes uploaded to Enterobase) and presence/absence of antimicrobial resistance (AMR) genes as detected by AMRFinderPlus coloured by AMR class, and presence/absence of plasmid replicons as detected by Abricate.

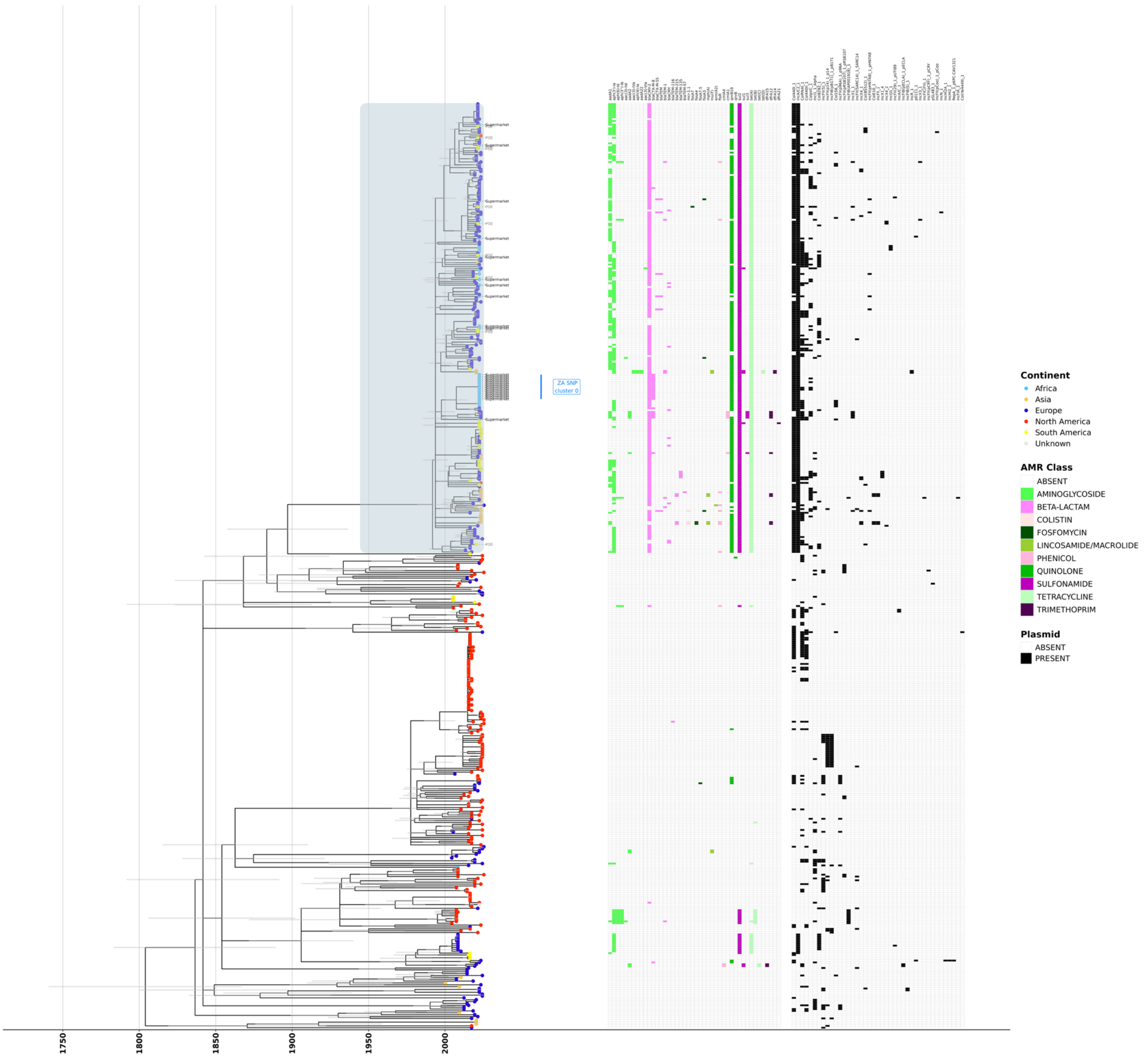

Fig S5: LSD2-estimated-rate maximum-likelihood (ML) phylogenetic tree constructed using IQ-TREE and RLSD2 comprising all 496 ST548 isolates.Tree tips are coloured by continent of isolation. Heatmap corresponding to tree tips indicate the source of the data (POE - Port of entry, Supermarket - poultry samples from supermarkets in South Africa, Public data - ST548 genomes uploaded to Enterobase) and presence/absence of antimicrobial resistance (AMR) genes as detected by AMRFinderPlus coloured by AMR class and presence/absence of plasmid replicons as detected by Abricate.

High quality versions of all figures are available in https://github.com/VishnuRaghuram94/SEPI/tree/main/manuscript_figs/png

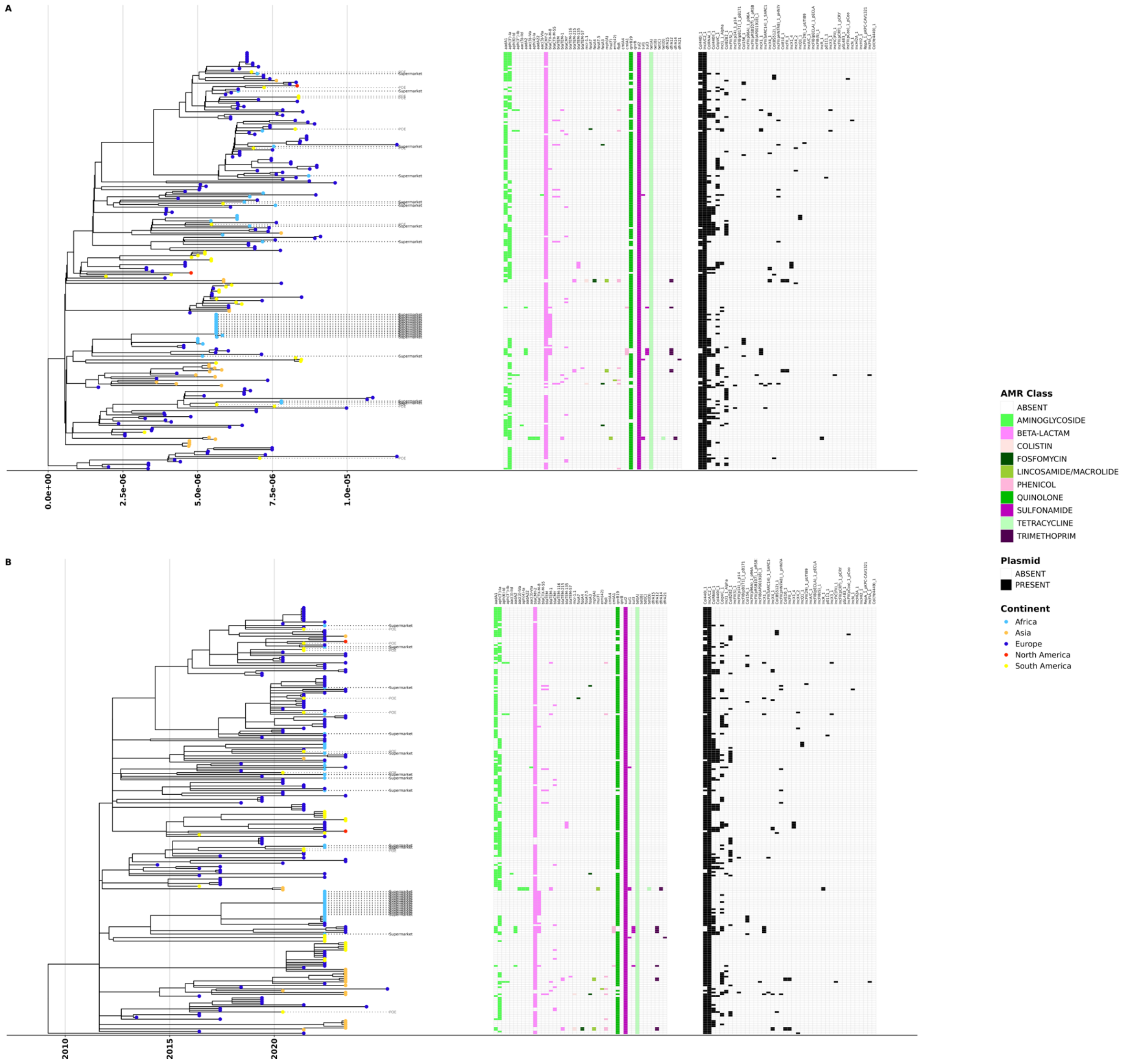

Fig S6: Midpoint rooted ML phylogeny (**A**) and (**B**) LSD2 rate-estimated time scaled phylogeny comprising the cluster of 241 high-AMR group isolates (this includes all novel isolates sequenced for this study). Tree tips are coloured by the continent of isolation. Heatmap corresponding to tree tips indicate presence/absence of antimicrobial resistance (AMR) genes as detected by AMRFinderPlus coloured by AMR class, and presence/absence of plasmid replicons as detected by Abricate.

High quality versions of all figures are available in https://github.com/VishnuRaghuram94/SEPI/tree/main/manuscript_figs/png

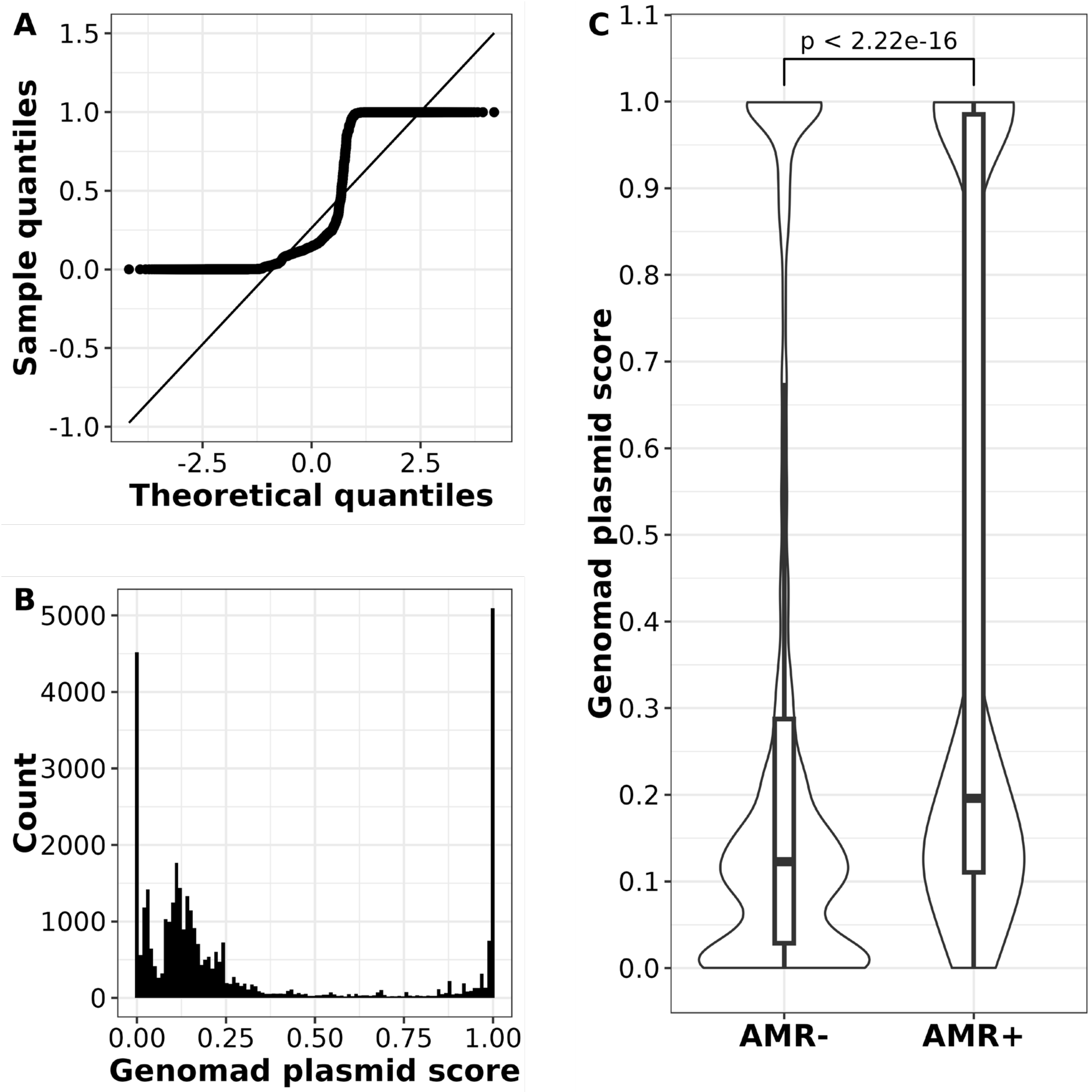

Fig S7: Quantile-quantile plot (**A**) and histogram (**B**) of GeNomad predicted plasmid scores of all assembled contigs from 496 ST548 genomes were observed to confirm non-normal distribution of data. Violin plot showing distribution of GeNomad predicted plasmid scores (y axis) for contigs not harbouring any AMR genes according to AMRFinderPlus (**AMR-** on x axis) vs contigs harbouring AMR genes (**AMR+** on x axis). Black horizontal line marks the mean. Kruskal Wallis test was performed comparing mean GeNomad plasmid scores between the two groups (AMR- vs AMR+).

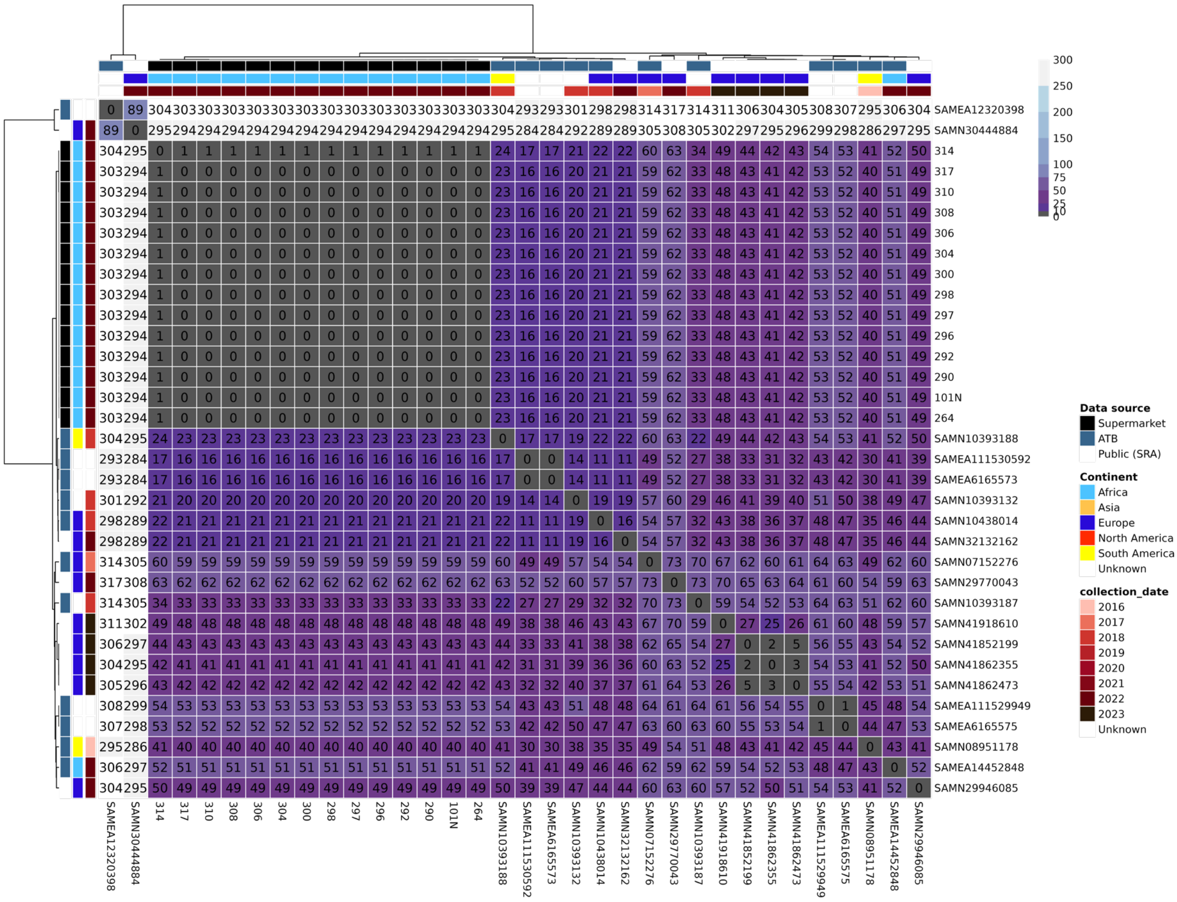

Fig S8: All vs all pairwise core SNP distance heatmap (lighter = greater SNP distance) of 34 *bla*$_{CTX\text{-}M\text{-}8}$+ isolates (14 isolates from 'ZA SNP cluster 0' plus 8 isolates from Enterobase plus 12 genomes from AllTheBacteria). Heatmap is annotated by 1) year of isolation; 2) continent of isolation and 3) Source of data (POE - Port of entry, Supermarket - poultry samples from supermarkets in South Africa, Public data - ST548 genomes uploaded to Enterobase) .

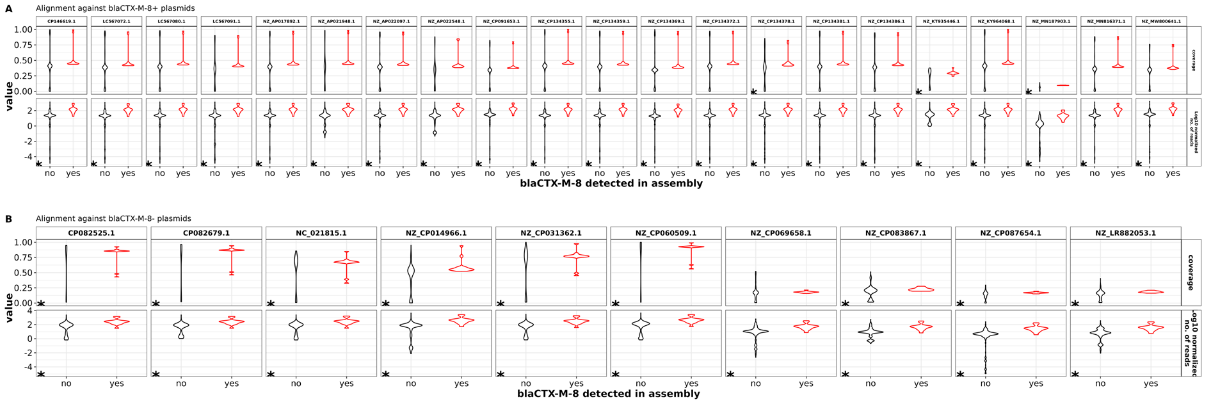

Fig S9: Violin plots comparing coverage and log10 transformed normalized number of mapped reads from *bla*$_{\text{CTX-M-8}}$+ genomes (red) and *bla*$_{\text{CTX-M-8}}$- genomes (black) when mapped to *bla*$_{\text{CTX-M-8}}$+ plasmids (**A**) and *bla*$_{\text{CTX-M-8}}$- plasmids (**B**) . Each facet represents the coverage or number of mapped reads against a specific plasmid, as denoted by the NCBI GenBank accession number in the facet header. “*” symbols in the bottom left corner of each facet, if present, represent statistically significant differences ($p < 0.01$; Wilcoxon test with FDR correction) for the corresponding metric (coverage or log10 transformed normalized number of mapped reads, as shown in the y axis facet labels on the right). Absence of “*” indicate non-significant differences ($p >= 0.01$)

High quality versions of all figures are available in https://github.com/VishnuRaghuram94/SEPI/tree/main/manuscript_figs/png

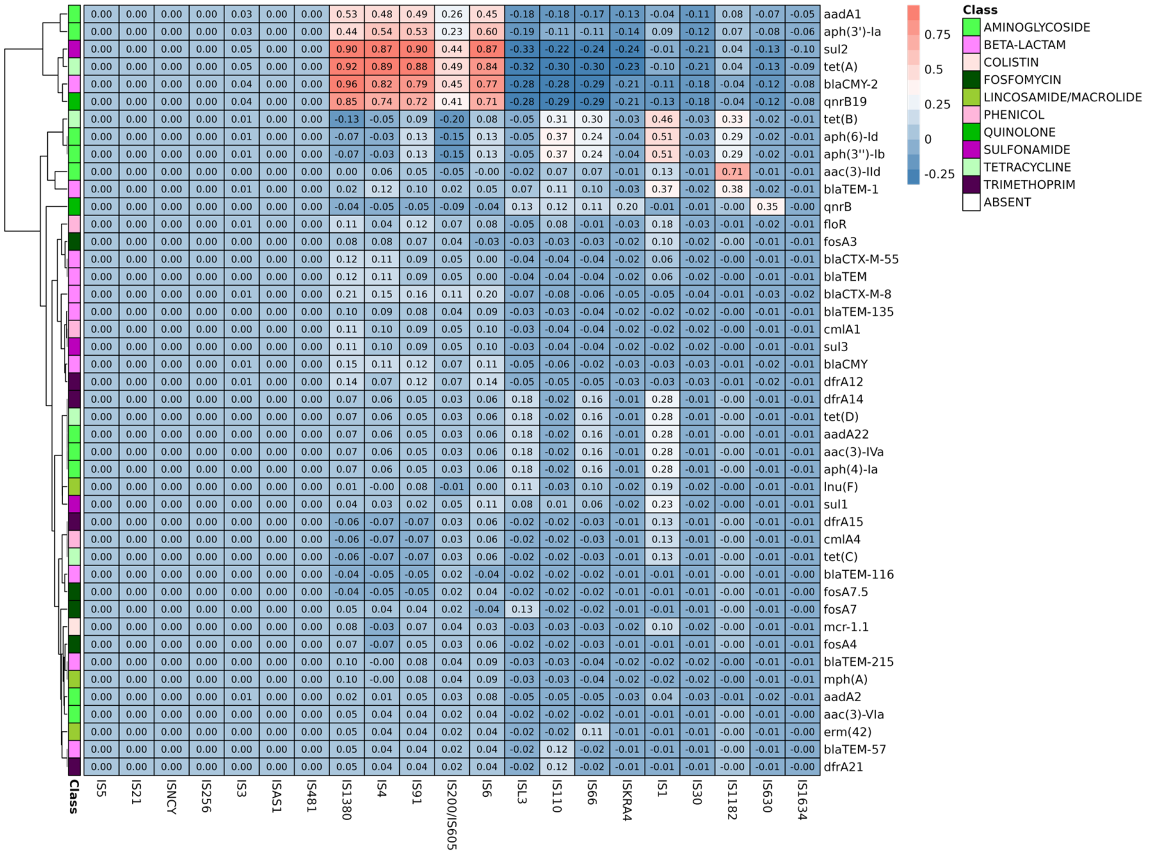

Fig S10: Heatmap showing Phi correlation between presence/absence of AMR gene (y-axis) vs presence/absence of IS elements (x-axis). Colours associated with AMR gene class are indicated on the left. The Phi-correlation value is indicated in each cell for the corresponding IS element - AMR gene pair.

Supplemental table legends:

Table S1: Internal sample names (`**sample_name**`), metadata (`**Collection year**`, `**Country**`, `**Continent**`, `**Data source**`, `**Isolation source**`), sequence type (`**ST**`**)** and NCBI accessions (`**biosample_accession**`, `**bioproject_accession**`, `**genome_acc**`) for 36 SEPI genomes + 460 public genomes analysed in this study (n=496).

Table S2: Novel isolates sequenced for this study (`**Ref_file**` column) and their corresponding closest publicly available isolate (`**Closest_public_sample**`). The core genome SNP distance between the novel and public isolates are in the `**Distance**` column. `**Collection Year**`,`**Country**`, `**Continent**` and `**Source Type**` columns show the year, country, continent and source of isolation of the corresponding public isolate. The biosample, bioproject and genome assembly accessions for the public isolate are in the `**biosample_accession**`,`**bioproject_accession**`,`**genome_acc**` columns respectively.

Table S3: Novel isolates sequenced for this study (**Ref_file** column) and their corresponding closest publicly available isolate from South Africa (**Closest_ZA_public_sample**). The core genome SNP distance between the novel and public isolates are in the `**Distance**` column. `**Collection Year**`,`**Country**`, and `**Source Type**` columns show the year, country (All from South Africa) and source of isolation of the corresponding public isolate.
